# Unconventional secretion of unglycosylated ORF8 is critical for the cytokine storm during SARS-CoV-2 infection

**DOI:** 10.1371/journal.ppat.1011128

**Published:** 2023-01-23

**Authors:** Xiaoyuan Lin, Beibei Fu, Yan Xiong, Na Xing, Weiwei Xue, Dong Guo, Mohamed Zaky, Krishna Pavani, Dusan Kunec, Jakob Trimpert, Haibo Wu

**Affiliations:** 1 School of Life Sciences, Chongqing University, Chongqing, China; 2 Institut für Virologie, Freie Universität Berlin, Berlin, Germany; 3 School of Pharmaceutical Sciences, Chongqing University, Chongqing, China; 4 Molecular Physiology Division, Faculty of Science, Beni-Suef University, Beni-Suef, Egypt; 5 Department of Reproduction, Obstetrics and Herd Health, Ghent University, Merelbeke, Belgium; University of Iowa, UNITED STATES

## Abstract

Coronavirus disease 2019 is a respiratory infectious disease caused by the severe acute respiratory syndrome coronavirus 2 (SARS-CoV-2). Evidence on the pathogenesis of SARS-CoV-2 is accumulating rapidly. In addition to structural proteins such as Spike and Envelope, the functional roles of non-structural and accessory proteins in regulating viral life cycle and host immune responses remain to be understood. Here, we show that open reading frame 8 (ORF8) acts as messenger for inter-cellular communication between alveolar epithelial cells and macrophages during SARS-CoV-2 infection. Mechanistically, ORF8 is a secretory protein that can be secreted by infected epithelial cells via both conventional and unconventional secretory pathways. Conventionally secreted ORF8 is glycosylated and loses the ability to recognize interleukin 17 receptor A of macrophages, possibly due to the steric hindrance imposed by N-glycosylation at Asn78. However, unconventionally secreted ORF8 does not undergo glycosylation without experiencing the ER-Golgi trafficking, thereby activating the downstream NF-κB signaling pathway and facilitating a burst of cytokine release. Furthermore, we show that ORF8 deletion in SARS-CoV-2 attenuates inflammation and yields less lung lesions in hamsters. Our data collectively highlights a role of ORF8 protein in the development of cytokine storms during SARS-CoV-2 infection.

## Author summary

Cytokine storm is an important point in the transition of COVID-19 from mild to severe/critical diseases. Clinical cases show that the viral loads in patients are not directly proportional to the severity of disease symptoms, which indicates that in addition to virion release, there is a unique indirect cellular communication mechanism in the development of cytokine storm. In this study, we found that accessory protein ORF8 is a secretory protein that acts as messenger for the inter-cellular communication between alveolar epithelial cells (the main targets of virus) and monocytes/macrophages (the main executors of cytokine storm). Our findings are helpful to understand the occurrence of cytokine storm caused by SARS-CoV-2, and provide a potential strategy for COVID-19 therapeutic drugs.

## Introduction

Coronavirus disease 2019 (COVID-19), caused by severe acute respiratory syndrome coronavirus 2 (SARS-CoV-2), is continuing to spread around the world with nearly 438 million confirmed cases and more than 5.9 million deaths. According to clinical case reports, a certain number of critically ill patients with COVID-19 experience cytokine storm, resulting in acute respiratory distress syndrome and multiple organ failure [1,2]. Although it has been reported that the mortality of COVID-19 patients is not necessarily related to the expression level of inflammatory factors [3,4], it is well-known that cytokine storm is an important point in the transition of COVID-19 from mild to severe diseases. Currently, our understanding of the mechanisms behind the cytokine storm and how SARS-CoV-2 affects cytokine release is still limited.

Open reading frame 8 (ORF8) is an accessory protein of SARS-CoV-2 and it is one of the most rapidly evolving β-coronaviruses proteins [5]. A 29 nucleotide deletion in ORF8 is the most obvious genetic change in severe acute respiratory syndrome coronavirus (SARS or SARS-CoV-1) during its host-jump from bats to humans [6]. The Δ382 variant of SARS-CoV-2, which eliminates ORF8 transcription, seems to be associated with milder infection and less systemic release of pro-inflammatory cytokines [7]. In a previous study, we demonstrated that ORF8 contributes to the cytokine storm during SARS-CoV-2 infection [8]. Specifically, we found ORF8 to interact with the interleukin 17 receptor A (IL17RA), leading to excessive activation of IL-17 signaling and downstream NF-κB pathway. However, it remains unclear how the virus exposes ORF8 to enable access to the extracellular domain of IL17RA.

In eukaryotes, secretory proteins usually contain a signal peptide that triggers translocation into the endoplasmic reticulum (ER) [9]. Following this translocation to the ER, cargoes will be exported through ER-Golgi trafficking for further processing and modification [10,11]. This process is termed conventional secretion. Besides, many cytosolic proteins without signal peptides, such as fibroblast growth factor 2 and yeast Acb1, can be released through an unconventional protein secretion pathway [12,13]. Evidently, viral proteins can hijack this secretion pathway to become secreted. For example, the HIV-1 Nef protein can be released from infected cells via an exosomal pathway [14,15]. However, very little is known about whether and how SARS-CoV-2 encoded proteins are secreted during infection.

Glycosylation is a common posttranslational modification, it involves the addition of glycans to macromolecules and is considered essential for the correct folding and functional performance of proteins [16–18]. It is also not uncommon for proteins from pathogens to be glycosylated by the host. The co-evolution of N-linked glycosylation sites in influenza viruses affects the host specificity [19]. The glycosylation of viral envelope proteins has a wide range of functions, including regulating cell tropism, protein stability and immune evasion [20–22]. Recent studies have shown that SARS-CoV-2 Spike protein has 22 N-linked glycosylation sites and 17 O-linked glycosylation sites, which may influence viral infectivity and pathogenicity [23–25]. In contrast to the situation for the Spike protein, glycosylation and its functional role in accessory proteins of SARS-CoV-2 has not yet been reported.

Here, we identified SARS-CoV-2 ORF8 as a secretory protein that can be secreted via conventional and unconventional secretory pathways at the same time. We found that unglycosylated ORF8 secreted via an unconventional pathway is responsible for the release of pro-inflammatory cytokines by binding the IL17RA receptor. By contrast, conventionally secreted ORF8 is incapable of binding to IL17RA due to the N-linked glycosylation at Asn78 site. Our findings present an important contribution to the understanding of how SARS-CoV-2 promotes the onset of cytokine storm, and provide a potential strategy for the development of COVID-19 therapeutics.

## Results

### SARS-CoV-2 ORF8 is a secretory protein that is associated with cytokine release

**Fig 1:**
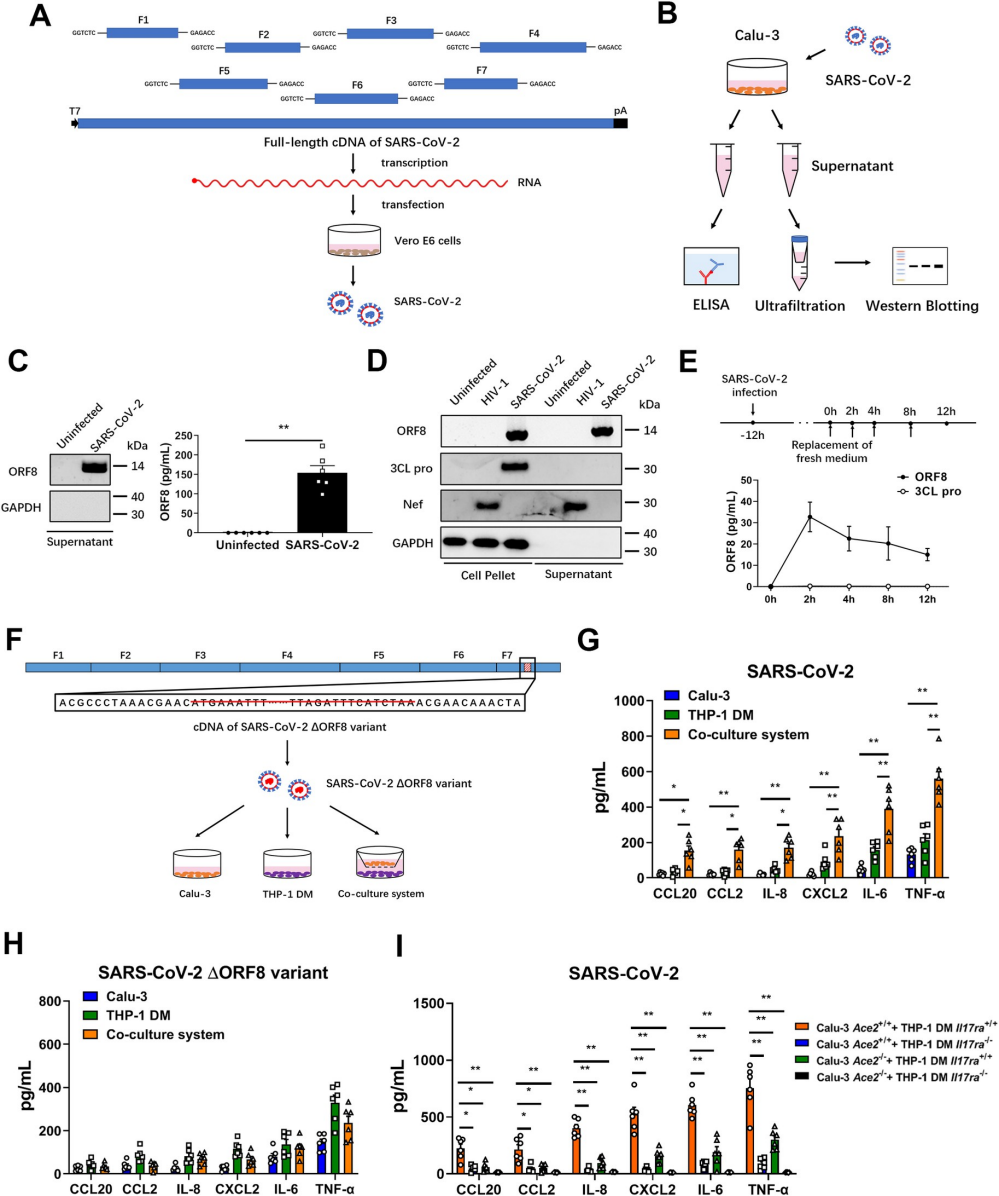
SARS-CoV-2 ORF8 can be secreted by epithelial cells. (A) Schematic diagram of SARS-CoV-2 infectious cDNA clone generated by a reverse genetic system. The cDNA fragments F1-F7 were synthesized and assembled into full-length SARS-CoV-2 cDNA, and RNA transcription, electroporation, and virus production were carried out in Vero E6 cells. (B) Schematic diagram of SARS-CoV-2 infection model. Calu-3 epithelial cells were infected with SARS-CoV-2 at a MOI of 0.01. After 12 hours, cell culture supernatant was centrifuged and divided into two parts for western blotting and ELISA, respectively. (C) The secretion of ORF8 in (B) was detected by western blotting and ELISA. (D) Jurkat cells or Calu-3 epithelial cells were infected with HIV-1 or SARS-CoV-2. After 12 hours, the secretion of ORF8, Nef and 3CL pro was detected by western blotting. (E) Schematic diagram of time-dependent ORF8 secretion upon SARS-CoV-2 infection. Twelve hours after SARS-CoV-2 infection, cell culture medium was replaced, and the amount of ORF8 protein in the supernatant was detected by ELISA every 2 hours. (F-H) Schematic diagram of ORF8-deleted SARS-CoV-2 variant (F). ORF8 coding sequence was deleted from the cDNA of F7 fragment. SARS-CoV-2 ΔORF8 variant was used to infect Calu-3 cells, THP-1 DM cells, and the co-culture system. The secretion of cytokines and chemokines related to cytokine storm was detected by ELISA (G, H). (I) Calu-3 *Ace2*^+/+^, Calu-3 *Ace2*^-/-^, THP-1 DM *Il17ra*^+/+^ and THP-1 DM *Il17ra*^-/-^ cells were used to form four kinds of culture systems. The secretion of cytokines and chemokines in different cell culture systems was detected by ELISA. Representative images from n = 3 biological replicates are shown (C, D). Data are shown as the mean ± s.e.m. of n = 6 biological replicates (C, E, G-I). Unpaired two-tailed Student *t* test (C) and one-way ANOVA followed by Bonferroni post *hoc* test (G, I) were used for data analysis. *, p < 0.05, **, p < 0.01.

In order to investigate the secretion of ORF8 protein, we infected Calu-3 human lung epithelial cells with SARS-CoV-2 that was generated using a reverse genetic system [26–28] (Fig 1A). Cell culture supernatant was collected and presence of ORF8 was determined by ELISA and western blotting (Fig 1B). We found that ORF8 protein can be secreted into the culture medium (Fig 1C). Then, we used four SARS-CoV-2 variants of concern isolated from COVID-19 patients, namely B.1.1.7 (Alpha), B.1.351 (Beta), B.1.1.28.1 (Gamma), and B.1.617.2 (Delta), to infect Calu-3 cells. The result confirmed that ORF8 secretion occurs in a physiologically relevant system (S1A Fig). To further validate this phenomenon, we used the non-secretory SARS-CoV-2 main proteinase (M pro, also known as 3CL pro) non-secreted control, and the well-known Nef secretory protein of HIV-1 [14,15] as a secreted protein control. We found that Jurkat cells secreted Nef protein following HIV-1 infection, and Calu-3 cells secreted ORF8 protein after being infected with SARS-CoV-2 (Fig 1D). By contrast, the non-structural protein 3CL pro was not secreted (Fig 1D). In order to test whether ORF8 is secreted through extracellular vesicles (EVs), we obtained EVs from culture medium by differential centrifugation, and found that ORF8 was barely existed in EVs (S1B Fig). In this study, we detected the release of ORF8 12 hours after SARS-CoV-2 infection. S1C Fig shows that Calu-3 cells did not undergo extensive apoptosis within 12 hours, excluding the possible involvement of apoptosis in ORF8 release. This result is consistent with a previous report demonstrating that the apoptosis of host cells occurs at least 24 hours post SARS-CoV-2 infection [29]. Next, we tested the time-dependency of ORF8 secretion in Calu-3 cells. After 12 hours of SARS-CoV-2 infection, the cell culture medium was replaced and ORF8 protein in the supernatant was detected every 2 hours. Using 3CL pro as a negative control, we found that the secretion of ORF8 continued for at least 12 hours after replacing the culture medium (Fig 1E). These results indicated that SARS-CoV-2 ORF8 is a secretory protein.

Our previous study has shown that ORF8 protein contributes to the cytokine storm during SARS-CoV-2 infection [8]. SARS-CoV-2 mainly invades alveolar epithelial cells through binding to ACE2 receptors, however monocytes/macrophages play a critical role in the secretion and regulation of cytokines. Next, we generated a SARS-CoV-2 variant with an ORF8 deletion (Fig 1F), and constructed an epithelial cell-macrophage co-culture system using a Transwell setup (Fig 1F), to answer the question that whether ORF8 is a key factor in modulating the transmission process of infection signals from epithelial cells to monocytes/macrophages. By measuring viral genomic RNA levels and titers, we firstly determined that ORF8 deletion did not affect the replication and infection of virus (S1D and S1E Fig). In this co-culture system, macrophages infected with wild-type SARS-CoV-2 or ORF8 deletion variant showed increased release of pro-inflammatory factors (Fig 1G and 1H), which is consistent with previous reports that macrophages are abortively infected but respond to SARS-CoV-2 by producing cytokines [30–32]. Interestingly, we found that the amount of pro-inflammatory factors in the co-culture system was much higher than that measured in individual epithelial cells or macrophages infected with wild-type SARS-CoV-2 (Fig 1G). However, this synergistic pro-inflammatory effect between epithelial cells and macrophages was not observed in the ORF8-deletion virus infected group (Fig 1H). Considering that ACE2 is responsible for virus entry [33], and IL17RA is the receptor of ORF8 [8,34], we generated ACE2-deficient epithelial cells (Calu-3 *Ace2*^-/-^) based on Calu-3 cell line, and IL17RA-deficient macrophages [THP-1-derived macrophages (THP-1 DM) *Il17ra*^-/-^] based on THP-1 (the rational for using human monocytic cell line is that pro-inflammatory cytokines are predominantly produced by monocytes/macrophages) (S1F Fig). The result of IFN-γ stimulation showed that IL17RA knockout THP-1 cells remain functional in terms of cytokine secretion (S1G Fig). By using these two ways to disrupt cellular communication between epithelial cells and macrophages, preventing SARS-CoV-2 entry by ACE2 deletion in Calu-3 *Ace2*^-/-^ cells, or interrupting ORF8 reception by IL17RA deletion in THP-1 DM *Il17ra*^-/-^ cells, a significant downregulation of pro-inflammatory factors was observed in the co-culture system (Fig 1I). These results implied that inter-cellular communication between epithelial cells and monocytes/macrophages is important for the cytokine release during SARS-CoV-2 infection.

It is known that secretory proteins, such as Nef, can be secreted in absence of viral infection in an *in vitro* system [14]. We therefore tested whether ORF8 protein can be secreted by human embryonic kidney (HEK-293FT) cells transfected with an ORF8-Flag plasmid. The results showed that Flag-tagged ORF8 was secreted in absence of SARS-CoV-2 infection (S1H and S1I Fig). Furthermore, the supernatant of cells transfected with ORF8-Flag was collected and added to the culture medium of THP-1 DM cells for stimulation. In this setup, the release of pro-inflammatory factors in THP-1 DM cells stimulated with ORF8-Flag transfection supernatant was significantly increased compared to cells treated with control supernatant (S1J Fig). In summary, we demonstrated that SARS-CoV-2 ORF8 is a secretory protein, and that extracellular ORF8 protein promotes cytokine release.

### SARS-CoV-2 ORF8 has an unconventional secretory pathway

**Fig 2:**
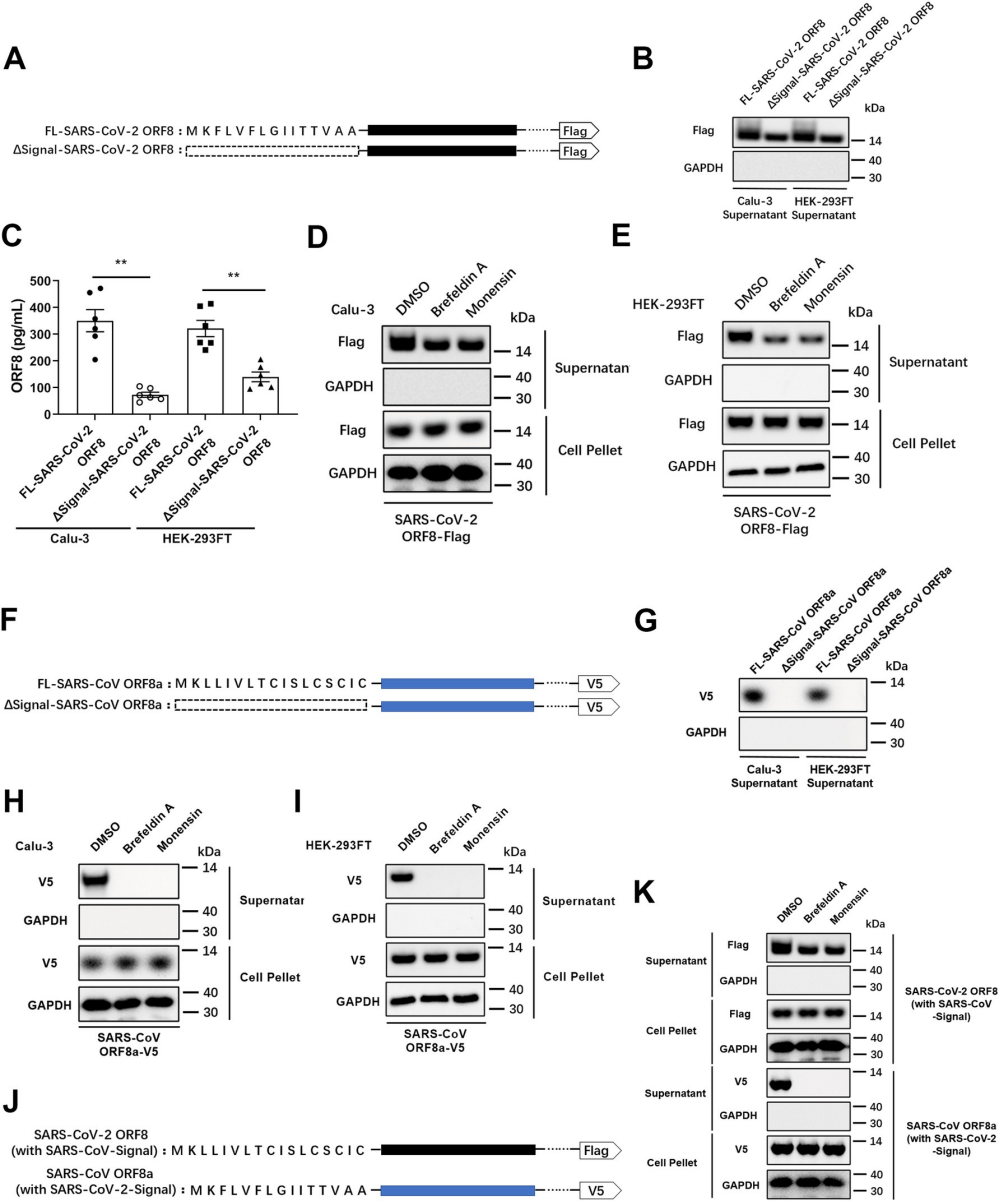
SARS-CoV-2 ORF8, rather than SARS-CoV ORF8, has an unconventional secretion pattern. (A) Structures of SARS-CoV-2 ORF8 and its signal peptide-deleted mutant. (B, C) Full-length SARS-CoV-2 ORF8 or its signal peptide-deleted mutant was transfected into Calu-3 cells or HEK-293FT cells. The secretion of ORF8 was detected by western blotting (B) and ELISA (C). (D, E) Brefeldin A (3 μg/mL) or Monensin (2 μM) was used to pretreat Calu-3 cells (D) or HEK-293FT cells (E) for 2 hours. Full-length SARS-CoV-2 ORF8 was transfected into pretreated cells. After 12 hours, the secretion of ORF8 was detected by western blotting. (F) Structures of SARS-CoV ORF8a and its signal peptide-deleted mutant. (G) Full-length SARS-CoV ORF8a or its signal peptide-deleted mutant were transfected into Calu-3 cells or HEK-293FT cells. The secretion of ORF8a was detected by western blotting. (H, I) Brefeldin A or Monensin was used to pretreat Calu-3 cells (H) or HEK-293FT cells (I) for 2 hours. Full-length SARS-CoV ORF8a was transfected into pretreated cells. After 12 hours, the secretion of ORF8a was detected by western blotting. (J) Structures of SARS-CoV-2 ORF8 mutant with signal peptide from SARS-CoV ORF8a (with SARS-Signal), and SARS-CoV ORF8a mutant with signal peptide from SARS-CoV-2 ORF8 (with SARS-CoV-2-Signal). (K) Brefeldin A or Monensin was used to pretreat Calu-3 cells for 2 hours. SARS-CoV-2 ORF8 with SARS-CoV-Signal and SARS-CoV ORF8a with SARS-CoV-2-Signal were transfected into pretreated Calu-3 cells. After 12 hours, the secretion of ORF8 was detected by western blotting. Representative images from n = 3 biological replicates are shown (B, D, E, G-I, K). Data are shown as the mean ± s.e.m. of n = 6 biological replicates (C). Unpaired two-tailed Student *t* test (C) was used for data analysis. **, p < 0.01.

In order to understand the secretion pattern of ORF8, we used an online tool, Simple Modular Architecture Research Tool (SMART, http://smart.embl-heidelberg.de), to analyze structural domains of the ORF8 protein. We found a hydrophobic central domain (similar to the conserved signal peptide of eukaryotes) to be located at its N-terminus. We defined this domain as the signal peptide of ORF8 protein. Signal peptide-deficient mutant (ΔSignal-SARS-CoV-2 ORF8) was constructed and transfected into Calu-3 epithelial cells and HEK-293FT cells, respectively (Fig 2A). The result showed that the secretion of ORF8 was significantly impaired by signal peptide deletion (Fig 2B and 2C). Interestingly, an appreciable quantity of signal peptide-deficient ORF8 was observed in both the supernatant of Calu-3 and HEK-293FT cells. This result strongly suggested that the secretion of ORF8 is not completely blocked by the signal peptide deletion (Fig 2B and 2C). This data implied that secretion of ORF8 might not completely depend on the presence of the signal peptide.

Proteins secreted through the conventional secretory pathway contain an N-terminal signal peptide, which is recognized by the signal recognition particles and transported into the ER, followed by signal peptide cleavage and trafficking to the Golgi apparatus and the subsequent endomembrane system [9]. To verify the existence of an unconventional secretory pathway for ORF8, Brefeldin A (a COPI inhibitor) [35] or Monensin [36] was used to inhibit the Golgi-related vesicle transportation. Consistent with our previous result, secretion of ORF8 was still observed when the conventional secretory pathway was blocked (Fig 2D and 2E). COPII vesicles are responsible for the export of secretory proteins from ER, which is the first step in the secretory pathway of mammalian cells. We used H89, an inhibitor that blocks COPII vesicle assembly at a high concentration [37], to determine the role of COPII vesicle in ORF8 secretion. The result showed that ORF8 secretion was abolished when export of cargo from the ER was prevented by COPII dissociation (S2 Fig). To understand whether the secretion pattern of ORF8 is evolutionary conserved, we analyzed the homologous ORF8 of SARS-CoV and found the SARS-CoV ORF8a isoform to contain an N-terminal signal peptide. We then constructed a ΔSignal-SARS-CoV ORF8a mutant and transfected it into epithelial cells (Fig 2F). While the intact SARS-CoV ORF8a was secreted normally, the ΔSignal-SARS-CoV ORF8a lost the secretory ability completely (Fig 2G). This finding stands in interesting contrast to our results showing that SARS-CoV-2 ORF8 is secreted in absence of the signal peptide (Fig 2G). This finding was further supported by inhibition of the Golgi-related vesicle transport using Brefeldin A or Monensin in Calu-3 (Fig 2H) and HEK-293FT cells (Fig 2I). The results obtained here show that SARS-CoV ORF8a can only be secreted under the guidance of signal peptide through the conventional secretory pathway.

Considering that the signal peptides of these two different ORF8 proteins share distinct sequences, we asked whether the different secretion patterns of ORF8 is due to the different types of signal peptide. To examine this hypothesis, we exchanged the ORF8 signal peptides between SARS-CoV and SARS-CoV-2 (Fig 2J). As a result, exchange of signal peptides did not change the secretory patterns observed for the two viruses (Fig 2K). Specifically, SARS-CoV-2 ORF8 carrying the SARS-CoV ORF8a signal peptide was still secreted when the Golgi-dependent secretory pathway was blocked (Fig 2K). Taken together, these results indicated that SARS-CoV-2 ORF8 is likely to have an unconventional secretion pattern that does not depend on the presence of a signal peptide.

### Unconventional secretion of ORF8 is required for cytokine storm

**Fig 3:**
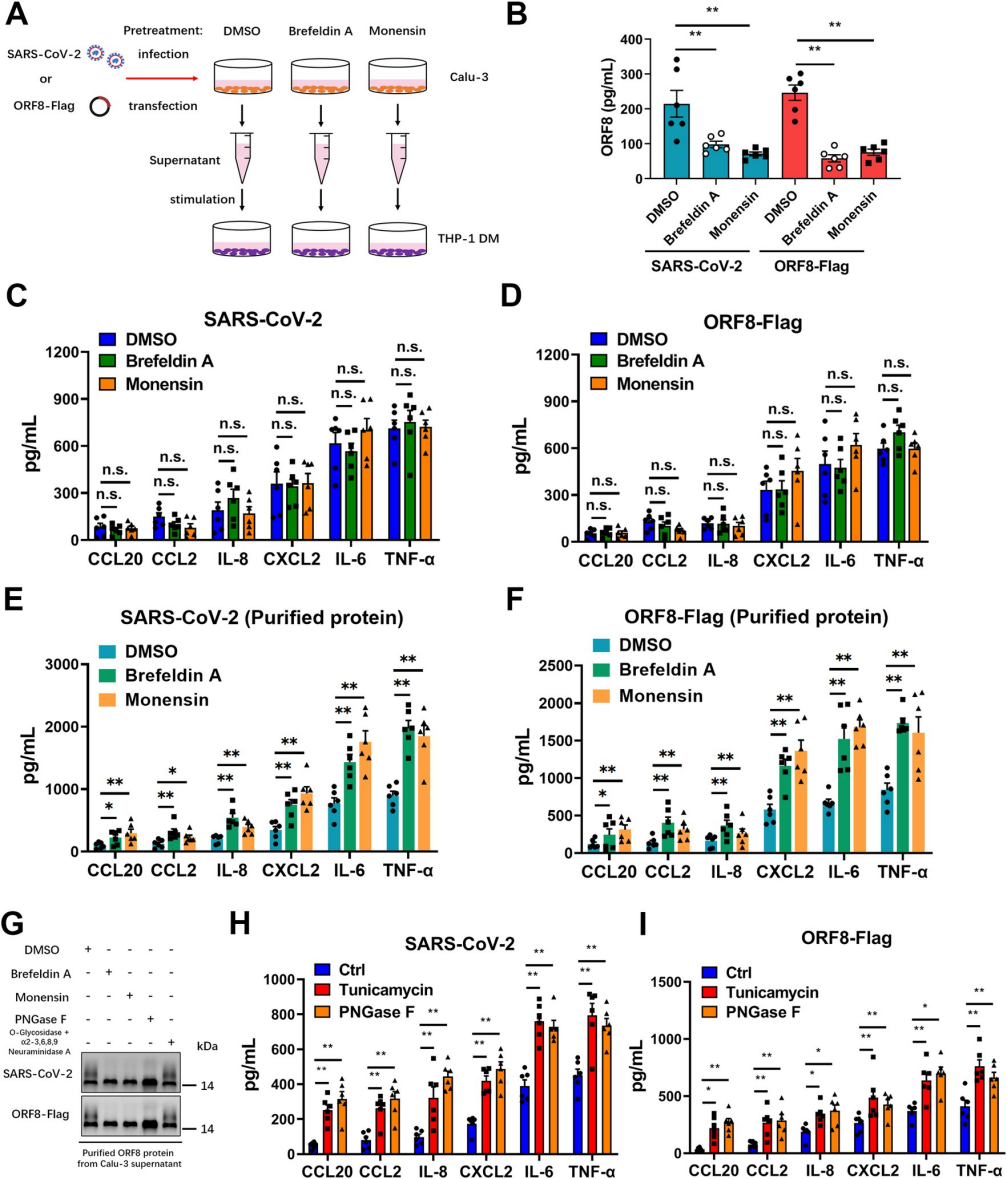
Unconventional secretion of ORF8 induces cytokine storm. (A) Schematic diagram of unconventional secretion model upon SARS-CoV-2 infection. Brefeldin A (3 μg/mL) or Monensin (2 μM) was used to pretreat Calu-3 cells for 2 hours, followed by SARS-CoV-2 infection, or Flag-tagged ORF8 plasmid transfection. After 12 hours, the supernatant was collected. (B-F) The supernatant collected in (A) was divided into three parts. One part was used for ELISA to examine the secretion of ORF8 protein (B); the second part was used to stimulate THP-1 DM cells (C, D); the third part was used to purify ORF8 protein and then stimulate THP-1 DM cells at a final concentration of 10 ng/mL (E, F). After 12 hours, the release of cytokines and chemokines was detected by ELISA (C-F). (G) Brefeldin A or Monensin was used to pretreat Calu-3 cells for 2 hours, followed by SARS-CoV-2 infection. ORF8 protein was purified 12 hours after infection and then PNGase F (1,000 units/μg protein), O-Glycosidase (4,000 units/μg protein) or α2–3, 6, 8, 9 Neuraminidase A (4 units/μg glycoprotein) was added to release glycans. Western blotting was used to detect the glycosylation of ORF8 protein. (H, I) Calu-3 cells were infected with SARS-CoV-2 (H) or transfected with ORF8-Flag plasmid (I). Tunicamycin (2μg/mL) was added into Calu-3 cells for 2 hours to prevent N-linked glycosylation; PNGase Fwas used to remove the N-linked glycosylation in purified ORF8 protein. After deglycosylation assays, purified ORF8 protein was used to stimulate THP-1 DM cells at a final concentration of 10 ng/mL. The release of cytokines and chemokines was detected by ELISA at 12 hours post stimulation. Representative images from n = 3 biological replicates are shown (G). Data are shown as the mean ± s.e.m. of n = 6 biological replicates (B-F, H, I). One-way ANOVA followed by Bonferroni post *hoc* test (B-F, H, I) was used for data analysis. *, p < 0.05, **, p < 0.01. Abbreviations: n.s., not significant.

Next, we studied whether the different secretion patterns of ORF8 are associated with the release of pro-inflammatory factors. Calu-3 epithelial cells were pretreated with Brefeldin A or Monensin to block the conventional secretory pathway, then cells were infected with SARS-CoV-2 and culture supernatant was collected to stimulate THP-1 DM cells. After 12 hours, pro-inflammatory factors secreted by macrophages were examined by ELISA (Fig 3A). We showed that Brefeldin A or Monensin treatment had no significant effect on viral replication (S3A Fig). Surprisingly, although the amount of secreted ORF8 was dramatically decreased (Fig 3B), the release of pro-inflammatory factors was barely affected by Brefeldin A or Monensin treatment (Fig 3C). In order to rule out the possibility of mutual influence between macrophages themselves, we tested pro-inflammatory factors released by THP-1 DM cells stimulated with supernatant at different time intervals. The result showed that there were no significant differences in the secretion of pro-inflammatory factors by macrophages within 0–12 h (Figs 3C and S3B). Consistent with previous results, we observed that the supernatant of THP-1 DM cells exogenously expressing Flag-tagged ORF8 induced almost equal levels of pro-inflammatory factors, regardless of the blockade of the Golgi-dependent secretory pathway (Figs 3D and S3C). Next, we used purified ORF8 protein from the supernatant after infection/transfection to further validate this finding. We showed that equal amounts of ORF8 protein purified from Brefeldin A or Monensin treated groups induced significantly higher levels of pro-inflammatory cytokines than that from the control group (Figs 3E, 3F, S3D and S3E). These results together implied that the unconventionally, instead of the conventionally secreted ORF8 is responsible for the cytokines release.

The current data point to a potential possibility that ORF8 secreted via different pathways might undertake different responsibilities. Interestingly, in our western blots, a smear band shifted to a higher molecular mass compared to ORF8 was regularly observed, this smear however disappeared when the conventional secretory pathway was inhibited by signal peptide deletion or Golgi apparatus damage (Fig 2B, 2D and 2E). Increasing the acrylamide concentration of the SDS-PAGE gel and prolonging the electrophoresis time, we were finally able to distinguish a second band of secreted SARS-CoV-2 ORF8 (Fig 3G). The Golgi apparatus is known to be the workshop for protein trafficking and processing [38], the most common form of protein processing in Golgi apparatus is glycosylation[17]. With this in mind, we asked whether ORF8 is glycosylated during the conventional secretory pathway, which might be responsible for the band shift. Using PNGase F or O-Glycosidase + α2–3, 6, 8, 9 Neuraminidase A, we tested the glycosylation status of ORF8 protein secreted from Calu-3 cells infected with SARS-CoV-2. We found that PNGase F treatment, which hydrolyzes most of the N-linked glycans [39,40], was leading to the formation of a single ORF8 protein band (Fig 3G). Digesting SARS-CoV-2 ORF8 with O-linked glycan hydrolase O-Glycosidase and α2–3, 6, 8, 9 Neuraminidase A did not change the type of bands compared to untreated samples (Fig 3G). These data suggested that part of the secreted SARS-CoV-2 ORF8 protein was N-glycosylated, and that this is likely a result of conventional secretion through the Golgi apparatus. By contrast, SARS-CoV ORF8a did not respond to glycoside hydrolases at all (S3F Fig), which means that conventionally secreted SARS-CoV ORF8a is not glycosylated.

Next, we asked whether the glycosylation status of ORF8 is associated with the release of pro-inflammatory factors. Calu-3 epithelial cells were infected with SARS-CoV-2, and tunicamycin [41] or PNGase F was used to oppose the N-linked glycosylation. Then purified ORF8 protein was added into the culture medium of THP-1 DM cells for stimulation. The results showed that macrophages stimulated with non-glycosylated ORF8 showed an elevated level of cytokine secretion (Figs 3H and S3G). Furthermore, we used plasmid transfection instead of SARS-CoV-2 infection to validate this result. In line with previous results, a similar upregulation in cytokine release was observed in the tunicamycin and PNGase F treatment groups (Figs 3I and S3H). Multiple measures at several time points showed that there were significant differences in cytokine levels between control group and the inhibitor treatment group from 6 h onwards (S3I and S3J Fig). These results indicated that the elevation of cytokines is a sustaining process. In contrast, the pro-inflammatory factor expression of Calu-3 cells transfected with SARS-CoV ORF8a-Flag plasmid did not change upon tunicamycin or PNGase F treatment (S3K Fig). Taken together, these results suggested that glycosylation state of ORF8 is closely associated with the release of pro-inflammatory factors.

### N-linked glycosylation at Asn78 impedes ORF8 binding to IL17RA

**Fig 4:**
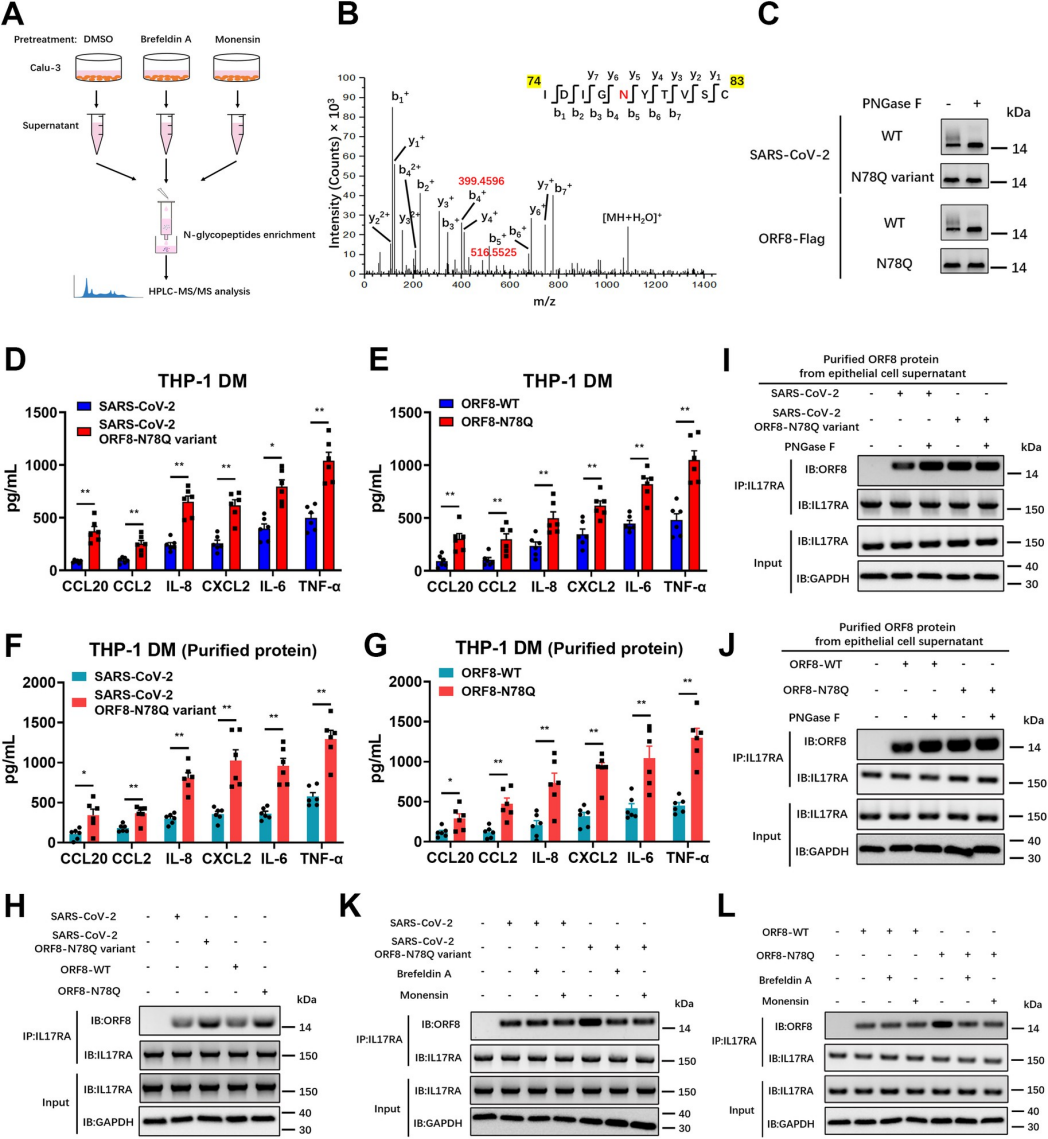
ORF8 N78 glycosylation blocks its interaction with IL17RA. (A) Schematic diagram of glycosylation identification based on HLPC-MS/MS. DMSO (control), Brefeldin A (3 μg/mL) or Monensin (2 μM) was used to pretreat Calu-3 cells for 2 hours. The supernatant was collected for HLPC-MS/MS analysis. (B) SARS-CoV-2 ORF8 secreted through conventional pattern has N78 glycosylation. An increase of 2.9890 Da of Asn residue was used to determine N-linked glycosylation. (C-G) Calu-3 cells were infected with SARS-CoV-2 ORF8-N78Q variant (C, D, F), or transfected with ORF8 N78Q plasmid (C, E, G). After 12 hours, the supernatant was collected and divided into three parts. One part was used to purify ORF8 protein, followed by PNGase F digestion and western blotting (C); the second part was used to stimulate THP-1 DM cells (D, E); the third part was used to purified ORF8 protein and then stimulate THP-1 DM cells at a final concentration of 10 ng/mL (F, G). After 12 hours, the release of cytokines and chemokines was detected by ELISA (D-G). (H) Calu-3 cells were infected with SARS-CoV-2 ORF8-N78Q variant, or transfected with ORF8-N78Q plasmid. Twelve hours later, the supernatant was used to stimulate THP-1 DM cells for another 12 hours. The interaction of ORF8 and IL17RA was detected by co-immunoprecipitation. (I, J) Calu-3 cells were infected with SARS-CoV-2 ORF8-N78Q variant (I), or transfected with ORF8-N78Q plasmid (J). After 12 hours, the supernatant was collected to purify ORF8 protein. After PNGase F digestion, the ORF8 protein was used to stimulate THP-1 DM cells for 12 hours, and the interaction of ORF8 and IL17RA was detected by co-immunoprecipitation. (K, L) Brefeldin A or Monensin was used to pretreat Calu-3 cells for 2 hours, followed by infection with SARS-CoV-2 ORF8-N78Q variant (K), or transfection with ORF8-N78Q plasmid (L). Twelve hours later, the supernatant was used to stimulate THP-1 DM cells for another 12 hours. The interaction of ORF8 and IL17RA was detected by co-immunoprecipitation. Representative images from n = 3 biological replicates are shown (C, H-L). Data are shown as the mean ± s.e.m. of n = 6 biological replicates (D-G). One-way ANOVA followed by Bonferroni post *hoc* test (D-G) was used for data analysis. *, p < 0.05, **, p < 0.01.

In order to determine the specific glycosylation site of SARS-CoV-2 ORF8, we collected the supernatant of SARS-CoV-2 infected Calu-3 epithelial cells and performed high performance liquid chromatography-tandem mass spectrometry (HPLC-MS/MS) for N-glycosylation site mapping (Fig 4A). According to the identification by MS, we found the Asparagine 78 (Asn78 or N78) of ORF8 protein to be glycosylated (Fig 4B). We further investigated the glycosylation site by creating a SARS-CoV-2 variant carrying the ORF8 N78Q mutation. By measuring viral RNA levels and titers, we found that N78Q mutation had no significant effect on the growth characteristics of the virus (S4A and S4B Fig). Following infection with a SARS-CoV-2 ORF8-N78Q mutant, Calu-3 epithelial cells secreted a single form of unglycosylated ORF8 (Fig 4C). This finding was also validated by transfection of exogenously expressed Flag-tagged N78Q ORF8 plasmid (Fig 4C). Further, we treated THP-1 DM cells with supernatant enriched from Calu-3 cells infected/transfected with wild-type or the SARS-CoV-2 ORF8-N78Q mutants, respectively. In this context, more pronounced cytokine release was observed in macrophages stimulated with the ORF8-N78Q mutants (Figs 4D, 4E, S4C and S4D). This result was further validated from the perspective of purified ORF8 protein. Consistent with previous results, ORF8 protein purified from the N78Q mutant groups led to the excessive expression of pro-inflammatory factors (Figs 4F, 4G, S4E and S4F). These data show that unglycosylated ORF8, instead of glycosylated ORF8, is involved in the inflammation response upon SARS-CoV-2 infection.

In our previous studies, we found that the interaction between ORF8 and host IL17RA contributes to the formation of a cytokine storm [8]. Here, we tested the effect of ORF8 glycosylation on the activation of the IL-17 pathway. We found that ORF8 protein secreted from the SARS-CoV-2 ORF8-N78Q variant or Flag-tagged ORF8-N78Q mutant, which could not be N-glycosylated at N78, exhibited stronger binding to the IL17RA receptor compared to a wild-type control (Fig 4H). Further, the interaction between SARS-CoV-2 ORF8 and IL17RA was significantly increased when PNGase F was used to remove glycosylation (Fig 4I). However, ORF8-N78Q showed consistently strong binding to IL17RA regardless of PNGase F treatment (Fig 4I). We also tested the activation of NF-κB signaling downstream of the IL-17 pathway. Consistently, the activation of NF-κB signaling was positively correlated with the binding affinity between ORF8 and IL17RA (S4G and S4H Fig). Data collected from plasmid transfection of Flag-tagged ORF8 mutant further validated this result (Figs 4J and S4I). To further confirm that the N-linked glycosylated ORF8 was secreted via a conventional pathway, Brefeldin A or Monensin was used to pretreat Calu-3 epithelial cells to block conventional secretory transport. As a result, inhibition of Golgi-dependent vesicle transport decreased the interaction between ORF8 and IL17RA in N78Q groups, because less ORF8 was secreted when ER-Golgi trafficking was blocked (S4J and S4K Fig); however, this inhibition did not affect the interaction between ORF8 and IL17RA in control groups, mainly because conventionally secreted ORF8 was glycosylated (Figs 4K, 4M, S4L and S4M). Additionally, we tested the effect of SARS-CoV ORF8a on the activation of IL-17 pathway. The results showed that SARS-CoV ORF8a was able to bind to the IL17RA receptor and activate the downstream NF-κB signaling (S4N and S4O Fig).

### ORF8 is essential for the onset of inflammatory responses

**Fig 5:**
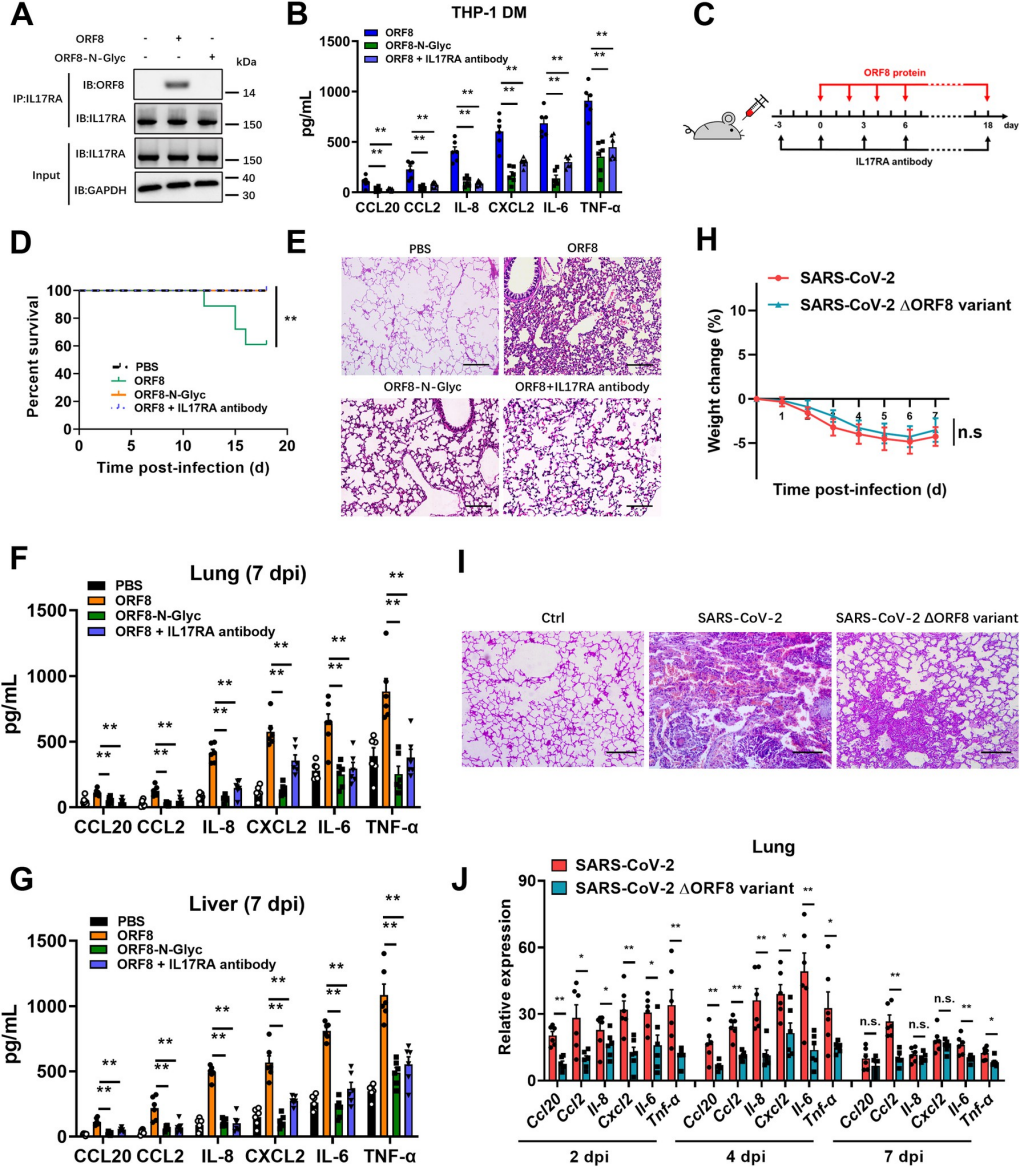
N-linked glycosylation of ORF8 protects mice from cytokine storm. (A) Unglycosylated ORF8 (ORF8) or synthetic N-linked-glycosylated ORF8 (ORF8-N-Glyc) (1 μg/mL) was added into the culture medium of THP-1 DM cells to stimulate IL-17 pathway. The interaction of ORF8 and IL17RA was detected by co-immunoprecipitation. (B) ORF8 or ORF8-N-Glyc protein was added into the culture medium of THP-1 DM cells at a concentration of 10 ng/mL. The release of cytokines and chemokines was detected by ELISA. (C) Schematic diagram of ORF8 protein treatment model. Mice were treated with aerosols of PBS (n = 13), unglycosylated ORF8 (n = 18) or synthetic N-linked-glycosylated ORF8 proteins (n = 17) (200 μg/mouse) every other day. For IL17RA antibody injections (n = 12), each mouse received 200 μg per injection every three days as indicated. (D-G) Survival curves were calculated using the Kaplan-Meier method (D). Lung lesions were detected by H&E staining at day 7 post infection (dpi) (E). The release of cytokines and chemokines in lungs (F) and livers (G) were detected by ELISA at 7 dpi. (H-J) Hamsters were intranasally infected with 4×10^5^ PFU SARS-CoV-2 (n = 18) or ORF8-deleted SARS-CoV-2 variant (n = 18). Weight loss data were documented daily (H). Lung lesions were detected by H&E staining at 7 dpi (I). The relative expression of cytokines and chemokines in lungs were detected by qRT-PCR at 2, 4 and 7 dpi (J). Representative images from n = 3 biological replicates are shown (A, E, I). Data are shown as the mean ± s.e.m. of n = 6 biological replicates (B, F, G, J). Log-rank (Mantel-Cox) test (D) and one-way ANOVA followed by Bonferroni post *hoc* test (B, F, G, J) were used for data analysis. Scale bar = 500 μm. *, p < 0.05, **, p < 0.01. Abbreviations: n.s., not significant.

Further, we prepared N-glycosylated ORF8 protein (ORF8-N-Glyc) *in vitro* and stimulated THP-1 DM cells directly. The results showed that glycosylated ORF8 could not bind the IL17RA receptor (Fig 5A), and that secretion of pro-inflammatory factors was significantly reduced (Fig 5B). These data indicated that glycosylation-deficient ORF8 is capable of binding to IL17RA and subsequent activation of the IL-17 pathway, thus promoting the cytokine release. In order to further verify the contribution of ORF8 glycosylation to the cytokine release and pathogenesis *in vivo*, we treated humanized ACE2 (hACE2) mice with aerosols of synthetic ORF8, ORF8-N-Glyc, or ORF8 and IL17RA antibody as indicated (Fig 5C). We observed a significant increase in weight loss and mortality in mice treated with unglycosylated ORF8 protein (Figs 5D and S5A). On the contrary, all the mice exposed to ORF8-N-Glyc, and mice treated with ORF8 and IL17RA antibody survived and exhibited no weight loss (Figs 5D and S5A). We also observed a mild inflammation in the lungs from mice treated with ORF8-N-Glyc, while the lung lesions in unglycosylated ORF8-exposed mice were much more severe (Figs 5E and S5B). Additionally, mice treated with ORF8-N-Glyc secreted decreased levels of cytokines and chemokines in lungs and livers when compared with the unglycosylated ORF8 groups, and treatment with IL17RA antibody could protect organs from unglycosylated ORF8-induced inflammation and damage (Figs 5F, 5G, S5C and S5D). Taken together, these data indicated that the N78 glycosylation of ORF8 participates in the regulation of host inflammatory responses.

To further characterize the functional role of ORF8 in the development of cytokine storm during SARS-CoV-2 infection, hamsters were intranasally infected with 4×10^5^ plaque-forming units (PFU) of ORF8 deletion variant. We showed that hamsters receiving the ORF8 deletion variant exhibited similar weight loss compared to the controls (Fig 5H); however, the lungs showed relieved inflammation compared to the extensive lung lesions observed in the control group (Figs 5I and S5E). Further, we examined the viral loads and cytokine release in the lungs of hamsters infected with ORF8 deletion variant. In this assay, although the viral genomic RNA levels and viral titers showed no significant difference between the two groups (S5F and S5G Fig), the cytokine release in the lungs of hamsters infected with ORF8 deletion variant was significantly alleviated compared to the control group (Fig 5J). These results emphasize the functional role of ORF8 protein in the development of a cytokine storm during SARS-CoV-2 infection.

**Fig 6:**
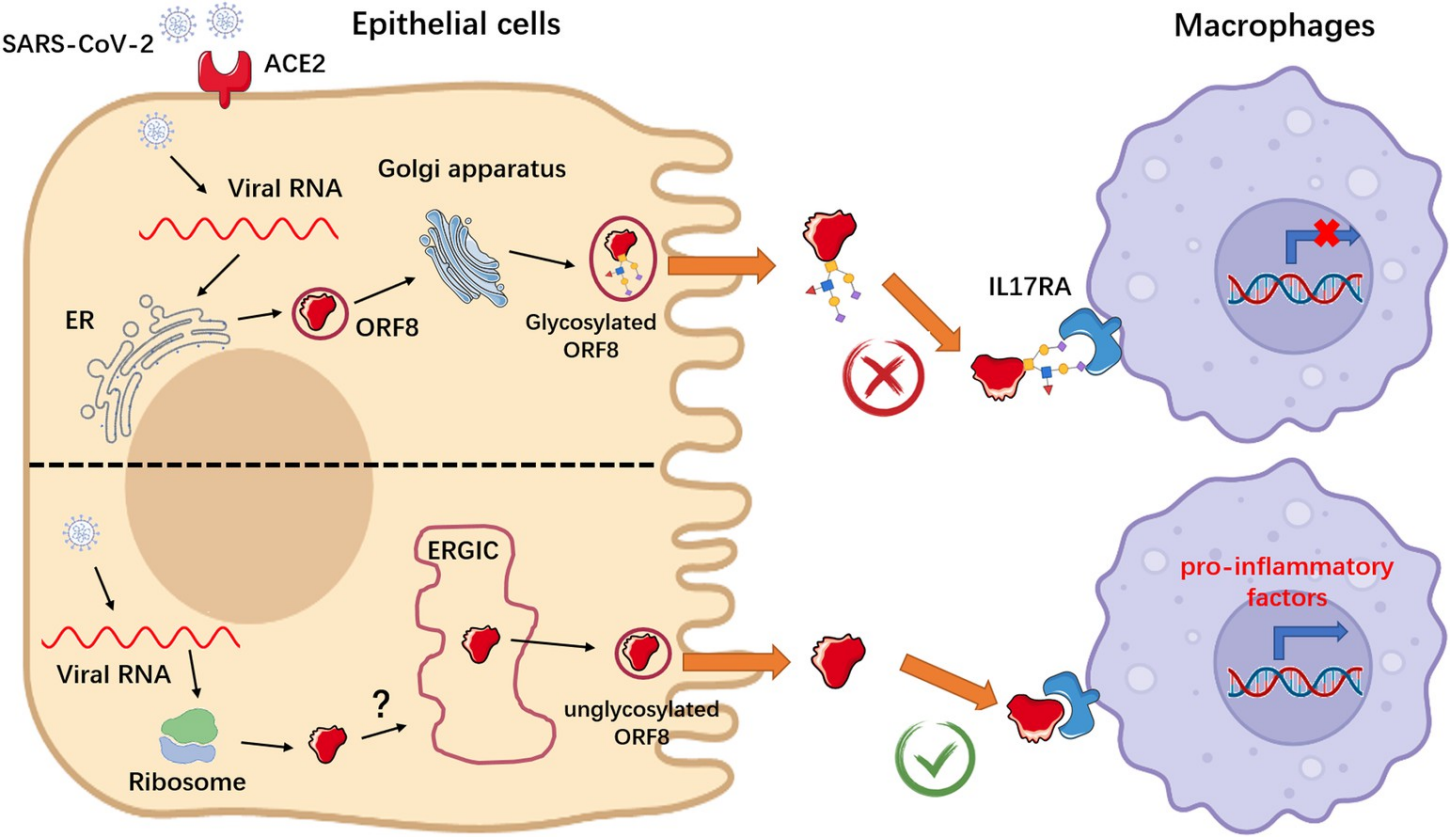
Schematic representation of conventional and unconventional secretion of ORF8 during SARS-CoV-2 infection.

Overall, we found that after invading host epithelial cells, SARS-CoV-2 ORF8 can be secreted through both conventional and conventional secretory pathways. In the conventional secretory pathway, ORF8 is N-glycosylated during the ER-Golgi trafficking, consequently, extracellular ORF8 lost the ability to recognize the IL17RA receptor of macrophages, likely due to steric hindrance imposed by N-glycosylation at the Asn78 site. By contrast, unconventionally secreted ORF8 protein does not become glycosylated without undergoing the conventional ER-Golgi trafficking. Hence, extracellular ORF8 can be distributed through body fluid circulation and to get in contact with macrophages, where unglycosylated ORF8 binds the IL17RA receptor and activates the IL17 pathway and downstream NF-κB signaling facilitating the onset of a cytokine storm (Fig 6).

## Discussion

Understanding the specific functions of SARS-CoV-2 proteins is pivotal for us to perceive the mechanisms underlying its high infectivity, fitness, and virulence. Numerous studies unravelling the differential functions of structural proteins have recently emerged [33,42]. However, it is worth noting that non-structural and accessory proteins encoded by SARS-CoV-2 likewise play significant roles in regulating the viral life cycle and influencing the host immune response. For example, ORF3a has been reported to induce apoptosis and promote lysosomal exocytosis-mediated viral egress [43,44], while ORF6 protein of SARS-CoV-2 hampers the induction of host interferon signaling [45].

ORF8, as a non-conserved accessory protein, is likely to be associated with the unique characteristics of SARS-CoV-2. According to clinical reports, ORF8 is highly immunogenic, with anti-ORF8 antibodies forming in the early stage of infection [46], and a significant T-cell response to ORF8 can be observed in recovered patients [47]. It was further reported that an ORF8-deficient SARS-CoV-2 strain (Δ382) in Singapore displays a significant reduction in virulence [7]. In a recent study, Zhang et al. reported that SARS-CoV-2 ORF8 interacts with MHC-I, a marker protein located on the cell surface, and activates the lysosomal degradation pathway, thus achieving escape from immune surveillance by decreasing the expression of MHC I [48]. These data indicate a specific role of ORF8 in infectivity and pathogenicity of SARS-CoV-2.

Our previous work has shown that SARS-CoV-2 ORF8 could interact with the IL17RA receptor, thereby leading to IL-17 pathway activation and an increased secretion of pro-inflammatory factors [8]. Considering that IL17RA is a transmembrane protein and ORF8 binds to the extracellular domain of IL17RA, as well as combining with the existing evidences, we proposed that ORF8 might be a secretory protein that is secreted into extracellular compartments. In this study, we show that SARS-CoV-2 ORF8 protein can be secreted by infected epithelial cells, which is supporting a role for ORF8 as a cellular messenger between alveolar epithelial cells and macrophages in the occurrence and development of a cytokine storm. Interestingly, we discovered that ORF8 can be secreted through both conventional and unconventional secretory pathways, and that ORF8 secreted via different pathways has different mechanistic functions in SARS-CoV-2 infection. Specifically, conventionally secreted ORF8 is N-glycosylated and loses the ability of binding the IL17RA receptor. Unconventionally secreted ORF8 does not undergo conventional ER-Golgi trafficking pathway and consequently is not glycosylated. It is therefore able to bind to the IL17RA receptor, activating IL-17 signaling and inducing the expression of pro-inflammatory factors. The existence of this unique indirect cellular communication mechanism during the course of SARS-CoV-2 infection, rather than virion release, may at least partly explain why viral loads in patients are not directly proportional to the severity of disease symptoms in COVID-19 [49,50].

In this study, we found that SARS-CoV ORF8a is also a secretory protein. Unlike SARS-CoV-2 ORF8, SARS-CoV ORF8a could only be secreted via a signal peptide-dependent conventional secretory pathway. Notably, the conventionally secreted SARS-CoV ORF8a is not glycosylated and is able to induce the release of pro-inflammatory factors. These differences might be due to the fact that ORF8 is the only SARS-CoV-2 protein with homology as low as approximate 20% to SARS-CoV [51]. In this context, the very different secretory pattern and functional role of ORF8 in SARS-CoV-2 could partly explain why the disease spectrum of COVID-19 differs from that of SARS. Based on our data, we propose a potential competition between host and guest regarding ORF8 protein: Primitive SARS invades host cells and secretes ORF8 through the conventional ER-Golgi trafficking pathway. ORF8 is not glycosylated in the Golgi apparatus and can induce the release of pro-inflammatory factors. However, the host does not just surrender to viral infection. During the evolutionary process, the host blocks the induction of IL-17 signaling though ORF8 glycosylation to maintain homeostasis. However, sarbecoviruses have evolved a new pathway to bypass glycosylation of ORF8 protein and secrete instead an unglycosylated form, thereby temporarily regaining an advantageous position in this competition. While triggering inflammation or tissue damage is not the ultimate goal of viral infection, this story provides a potential answer to the question of why the conventional secretion of ORF8 exists since signal peptide is not a determinant as the unconventional pathway could do the job. Nevertheless, the storyline needs to be validated by further studies.

Recently, several groups have independently reported that ORF8 is a secretory protein [34,52–55]; however, some of the results seem inconsistent with the others. For example, we found that the unglycosylated form of ORF8 was relatively low in Matsuoka’s system [52], while in our system 34.5~46.1% of ORF8 (Fig 3B) was unglycosylated. Considering the facts reported by Matsuoka et al. that wild-type ORF8 was abnormally expressed in 293T cells by transfection with expression plasmids and only the codon optimized ORF8 can be secreted extracellularly [52]. In contrast, in our system, secretion of ORF8 protein was routinely observed in Calu-3 human lung epithelial cells by infection with authentic SARS-CoV-2 virus/transfection with wild-type ORF8 plasmids. We speculate that this difference may be due to the different infection/expression systems and cell types. This hypothesis also applies to the study by Wu et al., who reported that ORF8-glyco^hi^ (purified ORF8 from the HEK-293 culture supernatant) induced inflammation, whereas ORF8-glyco^null^ (purified ORF8 from ORF8-expressing E. coli) did not. Oppositely, they found that although the glycosylation level was reduced by Brefeldin A and Monensin treatment, the cytokine-inducing activity of ORF8 was actually increased [53]. The second result supports our study; however it is contrary to their own conclusion that glycosylated ORF8 induces inflammation. We believe that the discrepancy in Wu’s study may be explained by that ORF8 purified from the HEK-293 culture supernatant contains an unglycosylated form, although the proportion should be low according to Matsuoka’s report. Moreover, Wang et al. reported that signal peptide is essential for extracellular secretion of ORF8 [56]; however, we found that ORF8 lacking a signal peptide could still be secreted, but in significantly reduced amount. It is based on this phenomenon that we discovered the unconventional secretion of ORF8. Taken together, the secretion of ORF8 protein is regulated by very sophisticated mechanisms. Different systems, including but not limited to infection/expression systems or cell types will affect the outcomes. Further in-depth and systematic studies should be performed to clarify the modification and secretion of ORF8 during SARS-CoV-2 infection. In addition, a substantial number of secreted eukaryotic proteins lacking classical signal peptides (called leaderless cargoes) are released through unconventional secretion. It has been reported that channel proteins located on the ER-Golgi intermediate compartment might mediate translocation of leaderless cargoes into transport vesicles. It would be interesting to further determine the channel protein that guides the translocation of ORF8 into an unconventional secretion pattern.

## Materials and methods

### Ethic statements

This study was carried out in strict accordance with the Guidelines for the Care and Use of Animals of Chongqing University. Animal experimental procedures were approved by the Laboratory Animal Welfare and Ethics Committee of Chongqing University. All hamster operations were performed under isoflurane anaesthesia to minimize animal pain. The SARS-CoV-2 live virus infection experiments were done under biosafety conditions in the BSL-3 facility at the Institut für Virologie, Freie Universität Berlin, Germany and performed in compliance with relevant institutional, national, and international guidelines for care and humane use of animal subjects.

### Cell lines and coronavirus

Calu-3 epithelial cells (HTB-55), Jurkat cells (Clone E6-1, TIB-152), THP-1 cells (TIB-202), Vero E6 cells (CRL-1586) and Sf9 cells (CRL-1711) were purchased from ATCC. HEK-293FT cells (R70007) were purchased from Thermo Fisher Scientific. Differentiation of THP-1 monocytes to macrophages was induced by 15 ng/mL phorbol 12-myristate 13-acetate (PMA) as previously described [57]. *Ace2*^-/-^ Calu-3 epithelial cell line and *Il17ra*^-/-^ THP-1-derived macrophage cell line were generated using CRISPR-Cas9 system with short guide RNA sequences (*Ace2*^-/-^ cell line: ACAGTTTAGACTACAATGAG; *Il17ra*^-/-^ cell line: TGTCCATTCGATGTGAGCCA. Murine alveolar epithelial cells (AECs) were isolated using the method developed by Corti and colleagues with motifications [58,59]. Plasmid and siRNA transfections were performed using a LONZA 4D-Nucleofector system according to the manufacturer’s instruction. The SARS-CoV-2 virus was generated by using a reverse genetic method as previously described [26–28]. Cells were infected with SARS-CoV-2 virus at a multiplicity of infection (MOI) of 0.01. Cell culture supernatant obtained from infected epithelial cells was filtered by Ultipor VF Grade UDV20 Virus Removal Filter Cartridges (PALL) to remove virion, and used for macrophage stimulation. Virus titer was determined using a standard TCID_50_ assay. For the generation of N78Q variant, AAT→CAA nucleotide substitutions were introduced into a subclone of pUC57-F7 containing the ORF8 gene of the SARS-CoV-2 wild-type infectious clone by overlap-extension PCR. Primers are as follows: F- TCAGTACATCGATATCGGTCAATAT, R- GTAAACAGGAAACTGTATATTGACC. SARS-CoV-2 variants B.1.1.7 (BetaCoV/Germany/ChVir21652/2020), B.1.351 (hCoV-19/Netherlands/NoordHolland_20159/2021), B.1.1.28.1 (hCoV-19/Netherlands/NoordHolland_10915/2021), and B.1.617.2 [SARS-CoV-2, Human, 2021, Germany ex India, 20A/452R (B.1.617)] [60] were grown in Vero E6 cells. The SARS-CoV-2 live virus infection experiments were done under biosafety conditions in the BSL-3 facility at the Institut für Virologie, Freie Universität Berlin, Germany.

### Animals and *in vivo* transfection

Specific-pathogen-free, six-week-old golden Syrian hamsters were purchased form Janvier Labs, and they were kept in individually ventilated cages. Hamsters were inoculated intranasally with 4×10^5^ PFU of SARS-CoV-2 virus. *In vivo* transfection was performed as previously described (26). Briefly, plasmid or siRNA was combined with *in vivo*-jetPEI delivery reagent (Polyplus-transfection, NY, USA) in a 10% glucose solution according to the manufacturer’s instruction. The solution was mixed and incubated for 30 min at room temperature, and then were intravenous injected into hamsters 12 hours before SARS-CoV-2 infection. Strengthen injection was performed at 3 dpi. Knockdown or overexpression efficiency was detected by western blotting at 2, 4 and 7 dpi.

### Quantification of viral genomic RNA

Orf1ab gene was used as the target site of primers for the quantification of viral genomic RNA. RT-PCR was performed with a SuperScript III One-Step RT–PCR kit and an ABI StepOnePlus PCR system (Applied Biosystems, CA, USA), according to the manufacturer’s instructions. Thermal cycling was performed at 50°C for 10 min for reverse transcription, followed by 95°C for 3 min and then 45 cycles of 95°C for 15 s, 58°C for 30s. The oligonucleotide sequences of the primers are as previously described (26).

### Collection of secretory proteins and immunoblot

Cell culture medium was collected and centrifuged twice to remove cell debris. The supernatant was concentrated through a 10 kDa Amicon-Ultra centrifugal tube (Millipore) and prepared for immunoblot. GAPDH was used to indicate the absence of cell lysates. Immunoblot analysis was performed as previously described [61]. Blots were probed with the indicated antibodies: anti-ORF8 (NBP3-05720), anti-GAPDH (NBP2-27103) (Novus Biologicals), anti-3CL pro (GTX135470) (GeneTex), anti-NEF (MA1-71507), anti-IL17RA (PA5-47199) (Thermo Fisher Scientific), anti-GFP (ab6556), anti-V5 (ab9137) (Abcam), anti-Flag (AF0036), anti-His (AF5060), anti-GST (AF5063), anti-HA (AF0039) (Beyotime Biotechnology, Shanghai, China).

### Enzyme linked immunosorbent assay

Cell culture supernatant was purified by centrifugation, and assayed by enzyme linked immunosorbent assays (ELISA). ORF8 antibody (NBP3-05720, Novus Biologicals) was coated with blank ELISA plates in carbonate buffer to prepare ELISA kit. Cytokine and chemokine ELISA kits were purchased from eBiosciences (Thermo Fisher Scientific).

### Block of conventional secretory pathway

Brefeldin A (00-4506-51, eBioscience, 3 μg/mL) or Monensin (00-4505-51, eBioscience, 2 μM) was added in the culture medium of epithelial cells for 2 hours to inhibit the ER-Golgi trafficking and block the ER-Golgi conventional secretion pathway.

### N-linked glycosylation identification by HPLC-MS/MS

N-linked glycosylation identification of SARS-CoV-2 ORF8 protein was performed as previously described with slight modifications [62]. Firstly, Calu-3 epithelial cells were infected by SARS-CoV-2 with/without Brefeldin A or Monensin pretreatment. The supernatant was collected and concentrated through a 10 kDa Amicon-Ultra centrifugal tube (Millipore). Then, the N-glycopeptides were enriched with Zic-HILIC (Fresh Bioscience), eluted and dried for deglycosylation. Enriched N-glycopeptides were digested using PNGase F dissolved in 50 mM NH_4_HCO_3_ (prepared with H_2_^18^O) for 2 hours at 37°C to remove N-linked glycosylation. Finally, deglycosylated peptides were dissolved in 0.1% FA for tandem mass spectrum analysis. MS1 was analyzed at an Orbitrap resolution of 120,000 using a scan range (m/z) of 800 to 2000 (N-glycopeptides before and after enrichment), or 350 to 1550 (deglycosylated peptides). The RF lens, AGC target, maximum injection time, and exclusion duration were 30%, 2.0 e^4^, 100 ms and 15 s, respectively. MS2 was analyzed with an isolation window (m/z) of 2 at an Orbitrap resolution of 15,000. The AGC target, maximum injection time, and the HCD type were standard, 250 ms, and 30%, respectively.

### Purification of ORF8 protein

Purification of ORF8 protein from the culture medium was performed using a Pierce Crosslink Magnetic IP/Co-IP Kit (88805, Thermo Fisher Scientific) according to the manufacturer’s instructions. Briefly, Protein A/G Magnetic Beads were prewashed twice with 1× Modified Coupling Buffer, followed by incubation with ORF8 antibody (NBP3-05720, Novus Biologicals) or Flag antibody (AF0036, Beyotime Biotechnology). Disuccinimidyl suberate was used for the linkage of antibodies to beads. Cell culture supernatant was concentrated and buffer exchanged using Amicon-Ultra centrifugal filters (Millipore), and then incubated with beads overnight at 4°C. ORF8 protein was collected using mild elution Buffer (Thermo Fisher Scientific). The purified ORF8 protein was aliquoted and stored at -80°C.

### Deglycosylation assays

Tunicamycin (12819, Cell Signaling Technology, 2μg/mL) was added into epithelial cells to prevent N-linked glycosylation. PNGase F (P0704, NEB, 1,000 units/μg protein) was used to remove the N-linked glycosylation in purified ORF8 protein. 1 μg glycoprotein, 2 μL GlycoBuffer 2 (10×), 2 μL PNGase F and H_2_O were mixed to form a 20 μL mixture. The mixture was incubated at 37°C for 8 hours, followed by western blotting analysis. To remove O-linked glycosylation, purified proteins were directly digested by O-glycosidase (P0733, NEB, 4,000 units/μg protein) and α2–3, 6, 8, 9 Neuraminidase A (P0722, NEB, 4 units/μg protein). 10 μg glycoprotein, 1 μL 10× Glycoprotein Denaturing Buffer and H_2_O were combined to make a 10 μL mixture. The glycoprotein was denatured by heating the mixture at 100°C for 10 min. Then, 2 μL 10× GlycoBuffer 2, 2 μL 10% NP40, 2 μL α2–3, 6, 8, 9 Neuraminidase A, 1 μL O-Glycosidase and H_2_O were mixed to form a total volume of 20 μL and incubated at 37°C for 4 hours, followed by western blotting analysis.

### Peptide synthesis and artificial glycosylation modification

SARS-CoV-2 ORF8 peptide was synthesized according to the NCBI published sequence (accession number: YP_009724396.1). Fmoc-L-Asn ((Ac)3-β-D-GlcNAc)-OH modification was performed by Shanghai Science Peptide Biological Technology Co., Ltd. ORF8 peptides with or without N-glycosylation were dissolved in 40 μL DMSO and diluted with PBS buffer for cell stimulation or mice aerosol infection.

### Histopathology analysis

Mice were anaesthetized with isoflurane, and lung lobes were harvested at indicated time points. Tissues were fixed with 10% PFA for more than 24 hours and embedded in paraffin. The paraffin blocks were cut into 2 μm-thick sections and stained using a standard Hematoxylin and eosin (H&E) procedure. Pathological changes of lung tissues were examined and evaluated by semiquantitative gross pathology scoring system as previously described [61].

### Statistical analysis

Sample size was based on empirical data from pilot experiments. The investigators were blinded during data collection and analysis. A value of P < 0.05 was considered significant.

## Supporting information

S1 Fig SARS-CoV-2 ORF8 can be secreted without the presence of complete virus. (A) Calu-3 epithelial cells were infected with SARS-CoV-2 variants of concern, including B.1.1.7 (Alpha), B.1.351 (Beta), B.1.1.28.1 (Gamma), and B.1.617.2 (Delta) at a MOI of 0.01. After 12 hours, the supernatants were collected to detect the secretion of ORF8. (B) Calu-3 epithelial cells were infected with SARS-CoV-2, or transfected with Flag-tagged ORF8. Exosomes were collected from culture medium for ORF8 detection at 12, 24 and 36 hours post infection. (C) Calu-3 epithelial cells were infected with SARS-CoV-2. Cell death rate was determined by Annexin V/PI staining and flow cytometry. (D, E) SARS-CoV-2 or SARS-CoV-2 ΔORF8 variant was used to infect Calu-3 cells at an initial MOI of 0.01. Genomic RNA (D) and viral titers (E) were detected at indicated time points. (F) Validation of Calu-3 *Ace2*^+/+^, Calu-3 *Ace2*^-/-^, THP-1 DM *Il17ra*^+/+^ and THP-1 DM *Il17ra*^-/-^ cells. The expressions of ACE2 and IL17RA were detected by western blotting. (G) THP-1 DM *Il17ra*^-/-^ cells were stimulated with recombinant human IFN-γ (10 ng/mL) for 12 hours. The release of cytokines was detected by ELISA. (H) Schematic diagram of THP-1 DM cells stimulation model. HEK-293FT cells were transfected with Flag-tagged ORF8, 3CL pro, or Nef. After 12 hours, the supernatant was collected and divided into two parts. One part was used to purify secretory proteins, followed by western blotting; the other part was used to stimulate THP-1 DM cells for 12 hours. The release of cytokines and chemokines was detected by ELISA. (I) Secretory proteins obtained from HEK-293FT cell supernatant in (G) were detected by western blotting. (J) The release of cytokines and chemokines from THP-1 DM cells in (G) was detected by ELISA. Representative images from n = 3 biological replicates are shown (A-C, F, I). Data are shown as the mean ± s.e.m. of n = 6 biological replicates (D, E, G, J). Unpaired two-tailed Student *t* test (D, E) and one-way ANOVA followed by Bonferroni post *hoc* test (G, J) were used for data analysis. **, p < 0.01. Abbreviations: n.s., not significant.
(TIF)

S2 Fig Blocking of COP II-coated vesicle assembly inhibits ORF8 secretion. (A) Calu-3 epithelial cells were treated with H89 (150 μM) to block COP II-coated vesicle assembly, followed by SARS-CoV-2 infection, or Flag-tagged ORF8 plasmid transfection. After 12 hours, the secretion of ORF8 was detected by western blotting. Representative images from n = 3 biological replicates are shown.
(TIF)

S3 Fig SARS-CoV ORF8a cannot be glycosylated. (A) Brefeldin A (3 μg/mL) or Monensin (2 μM) was used to pretreat Calu-3 cells for 2 hours, followed by SARS-CoV-2 infection at an MOI of 0.01. Cell lysates were harvested at indicated time points for detection of genomic RNA. (B-E) Brefeldin A or Monensin was used to pretreat Calu-3 cells for 2 hours, followed by SARS-CoV-2 infection (B, D), or Flag-tagged ORF8 plasmid transfection (C, E). After 12 hours, the supernatant was collected and divided into two parts. One part was used to stimulate THP-1 DM cells (B, C); the other part was used to purify ORF8 protein and then stimulate THP-1 DM cells at a final concentration of 10 ng/mL (D, E). The release of cytokines and chemokines was detected by ELISA at the indicated time points. (F) Brefeldin A or Monensinwas used to pretreat Calu-3 cells for 2 hours, followed by Flag-tagged SARS-CoV ORF8a plasmid transfection. ORF8a protein was purified and then PNGase F (1,000 units/μg protein), O-Glycosidase (4,000 units/μg protein) or α2–3, 6, 8, 9 Neuraminidase A (4 units/μg glycoprotein) was added to release glycans. Western blotting was used to detect the glycosylation of ORF8a protein. (G, H) The fold change analysis of cytokine and chemokine release in Fig 3H (G) and 3I (H). (I-K) Calu-3 cells were infected with SARS-CoV-2 (I), or transfected with ORF8-Flag (J) or SARS-CoV ORF8a-Flag plasmid (K). Tunicamycin (2μg/mL) was added into Calu-3 cells for 2 hours to prevent N-linked glycosylation; PNGase F was used to remove the N-linked glycosylation in purified ORF8 or ORF8a protein. After deglycosylation assays, purified ORF8 or ORF8a protein was used to stimulate THP-1 DM cells at a final concentration of 10 ng/mL. At the indicated time points, the release of cytokines and chemokines was detected by ELISA. Representative images from n = 3 biological replicates are shown (F). Data are shown as the mean ± s.e.m. of n = 6 biological replicates (A-E, G-K). Two-way ANOVA followed by Bonferroni post *hoc* test (A-E, G-K) was used for data analysis. *, p < 0.05, **, p < 0.01. Abbreviations: n.s., not significant.
(TIF)

S4 Fig ORF8 N78 glycosylation blocks the activation of IL-17 pathway. (A, B) SARS-CoV-2 or SARS-CoV-2 ORF8-N78Q variant was used to infect Calu-3 cells at an MOI of 0.01. Genomic RNA (A) and viral titers (B) were detected at indicated time points. (C-F) Calu-3 cells were infected with SARS-CoV-2 ORF8-N78Q variant (C, E), or transfected with ORF8 N78Q plasmid (D, F). After 12 hours, the supernatant was collected and divided into two parts. One part was used to stimulate THP-1 DM cells (C, D); the other part was used to purify ORF8 protein and then stimulate THP-1 DM cells at a final concentration of 10 ng/mL (E, F). The release of cytokines and chemokines was detected by ELISA at the indicated time points. (G) Calu-3 cells were infected with SARS-CoV-2 ORF8-N78Q variant, or transfected with ORF8-N78Q plasmid. The supernatant was used to stimulate THP-1 DM cells for 12 hours, the activation of IL-17 pathway was evaluated by testing NF-κB activity. (H, I) Calu-3 cells were infected with SARS-CoV-2 ORF8-N78Q variant (H), or transfected with ORF8-N78Q plasmid (I). The supernatant was collected to purify ORF8 protein. After PNGase F digestion, the ORF8 protein was used to stimulate THP-1 DM cells. After 12 hours, the activation of IL-17 pathway was evaluated by testing NF-κB activity. (J, K) Brefeldin A or Monensin was used to pretreat Calu-3 cells for 2 hours, followed by infection with SARS-CoV-2 ORF8-N78Q variant (H) or transfection with ORF8-N78Q plasmid (I). The supernatant was used to stimulate THP-1 DM cells for 12 hours. The secretion of ORF8 was detected by ELISA. (L, M) The activation of IL-17 pathway in (J, K) was evaluated by testing NF-κB activity. (N, O) Brefeldin A or Monensin was used to pretreat Calu-3 cells for 2 hours, followed by Flag-tagged SARS-CoV ORF8a plasmid transfection. ORF8a protein was purified and then PNGase F (1,000 units/μg protein), O-Glycosidase (4,000 units/μg protein) or α2–3, 6, 8, 9 Neuraminidase A (4 units/μg glycoprotein) was added to release glycans. Subsequently, ORF8a protein was used to stimulate THP-1 DM cells. After 12 hours, the interaction of ORF8a and IL17RA was detected by co-immunoprecipitation (N), and the activation of IL-17 pathway was evaluated by testing NF-κB activity (O). Representative images from n = 3 biological replicates are shown (N). Data are shown as the mean ± s.e.m. of n = 6 biological replicates (A-M, O). Unpaired two-tailed Student *t* test (A, B), two-way (C-F) and one-way ANOVA (G-M, O) followed by Bonferroni post *hoc* test were used for data analysis. *, p < 0.05, **, p < 0.01. Abbreviations: n.s., not significant.
(TIF)

S5 Fig Mice infected with glycosylated ORF8 show mild tissue injury. (A) Weight change of hACE2 mice treated with PBS, unglycosylated ORF8, synthetic N-linked-glycosylated ORF8 proteins, or unglycosylated ORF8 and IL17RA antibody. Data are shown as the mean ± s.e.m. (B) Histologic scoring of lungs obtained from hACE2 mice at 7 dpi in Fig 5E. (C, D) The release of cytokines and chemokines in lungs (C) and livers (D) were detected by ELISA at 14 dpi. (E) Histologic scoring of lungs obtained from hamsters at 7 dpi in Fig 5I. (F) Viral genomic RNA in nasal wash, trachea and lungs obtained from hamsters infected with SARS-CoV-2 or ORF8-deleted SARS-CoV-2 variant at 2, 4 and 7 dpi were detected by qRT-PCR. (G) Viral titers in lungs obtained from hamsters infected with SARS-CoV-2 or ORF8-deleted SARS-CoV-2 variant at 2, 4 and 7 dpi were detected by TCID_50_ assay. LOD, limit of detection. Data are shown as the mean ± s.e.m. of n = 6 biological replicates (B-G). One-way ANOVA followed by Bonferroni post *hoc* test was used for data analysis. *, p < 0.05, **, p < 0.01. Abbreviations: n.s., not significant.
(TIF)
