# Supplementary figures and images for: Unconventional secretion of unglycosylated ORF8 is critical for the cytokine storm during SARS-CoV-2 infection
Source: PLoS Pathog. 2023 Jan 23;19(1):e1011128. doi: 10.1371/journal.ppat.1011128 (PMC9894554; doi:10.1371/journal.ppat.1011128)

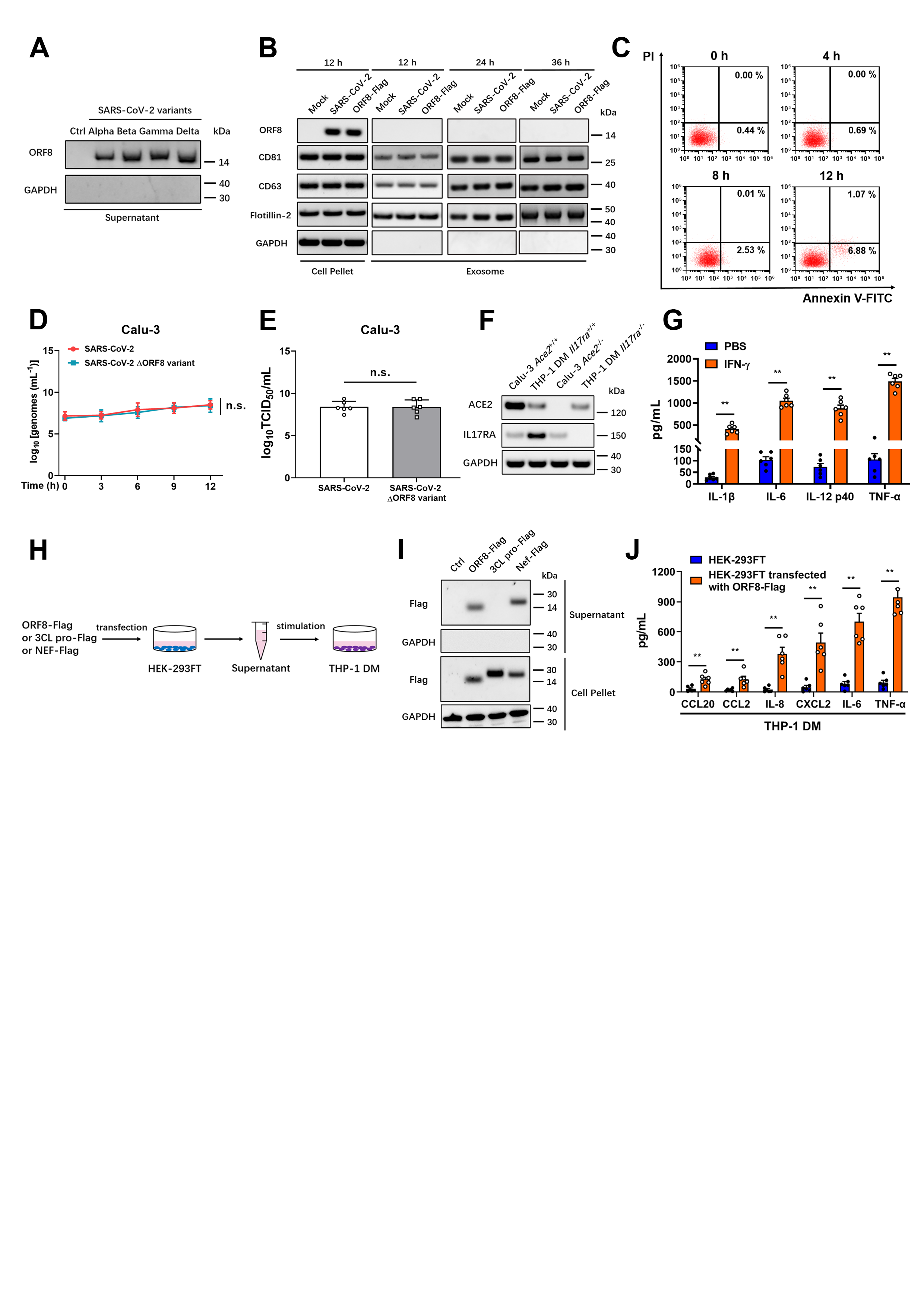

Supplement: S1 Fig — (A) Calu-3 epithelial cells were infected with SARS-CoV-2 variants of concern, including B.1.1.7 (Alpha), B.1.351 (Beta), B.1.1.28.1 (Gamma), and B.1.617.2 (Delta) at a MOI of 0.01. After 12 hours, the supernatants were collected to detect the secretion of ORF8. (B) Calu-3 epithelial cells were infected with SARS-CoV-2, or transfected with Flag-tagged ORF8. Exosomes were collected from culture medium for ORF8 detection at 12, 24 and 36 hours post infection. (C) Calu-3 epithelial cells were infected with SARS-CoV-2. Cell death rate was determined by Annexin V/PI staining and flow cytometry. (D, E) SARS-CoV-2 or SARS-CoV-2 ΔORF8 variant was used to infect Calu-3 cells at an initial MOI of 0.01. Genomic RNA (D) and viral titers (E) were detected at indicated time points. (F) Validation of Calu-3 Ace2+/+, Calu-3 Ace2-/-, THP-1 DM Il17ra+/+ and THP-1 DM Il17ra-/- cells. The expressions of ACE2 and IL17RA were detected by western blotting. (G) THP-1 DM Il17ra-/- cells were stimulated with recombinant human IFN-γ (10 ng/mL) for 12 hours. The release of cytokines was detected by ELISA. (H) Schematic diagram of THP-1 DM cells stimulation model. HEK-293FT cells were transfected with Flag-tagged ORF8, 3CL pro, or Nef. After 12 hours, the supernatant was collected and divided into two parts. One part was used to purify secretory proteins, followed by western blotting; the other part was used to stimulate THP-1 DM cells for 12 hours. The release of cytokines and chemokines was detected by ELISA. (I) Secretory proteins obtained from HEK-293FT cell supernatant in (G) were detected by western blotting. (J) The release of cytokines and chemokines from THP-1 DM cells in (G) was detected by ELISA. Representative images from n = 3 biological replicates are shown (A-C, F, I). Data are shown as the mean ± s.e.m. of n = 6 biological replicates (D, E, G, J). Unpaired two-tailed Student t test (D, E) and one-way ANOVA followed by Bonferroni post hoc test (G, J) were used for data analysis. [file ppat.1011128.s001.tif]

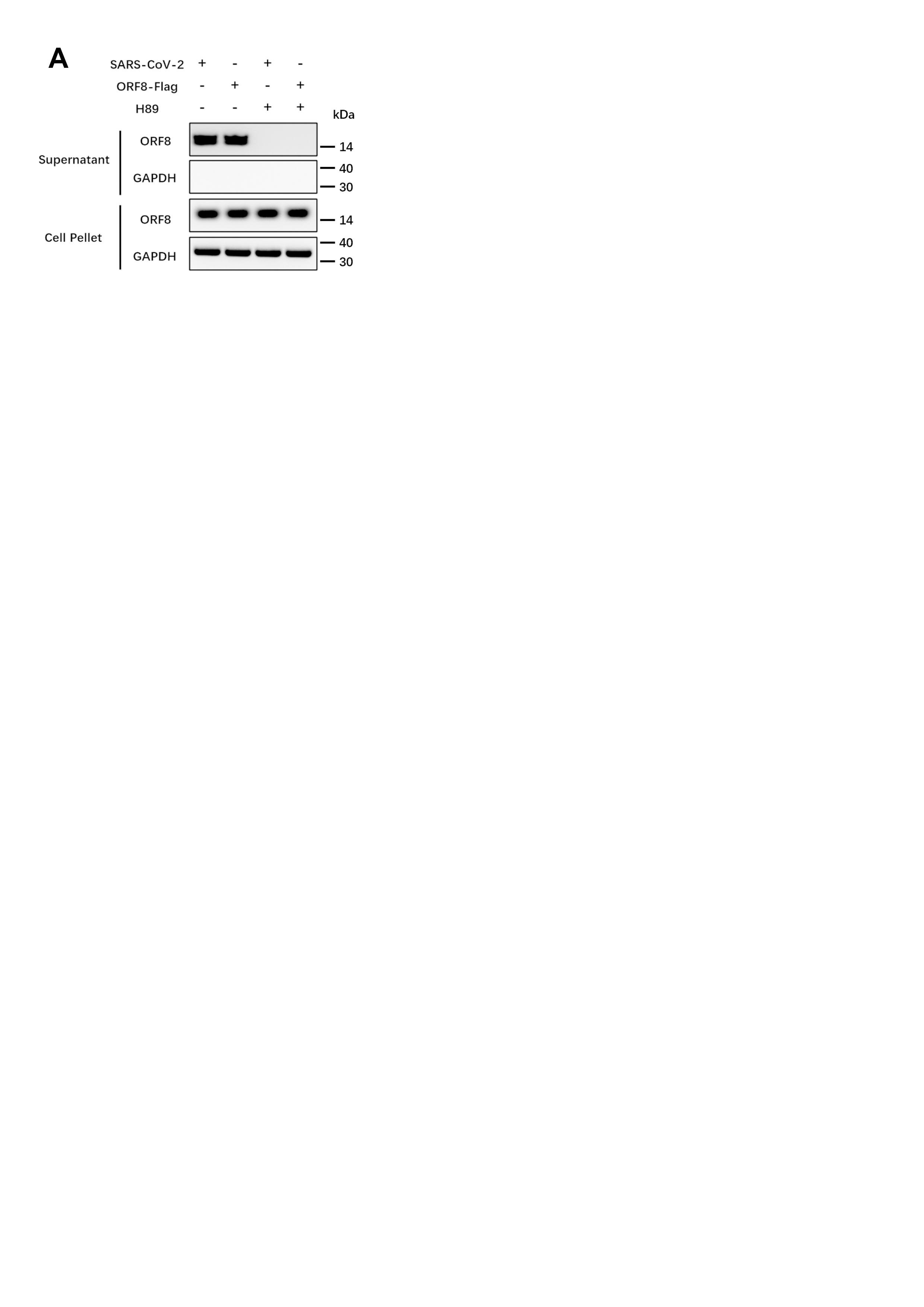

Supplement: S2 Fig — (A) Calu-3 epithelial cells were treated with H89 (150 μM) to block COP II-coated vesicle assembly, followed by SARS-CoV-2 infection, or Flag-tagged ORF8 plasmid transfection. After 12 hours, the secretion of ORF8 was detected by western blotting. Representative images from n = 3 biological replicates are shown. (TIF) [file ppat.1011128.s002.tif]

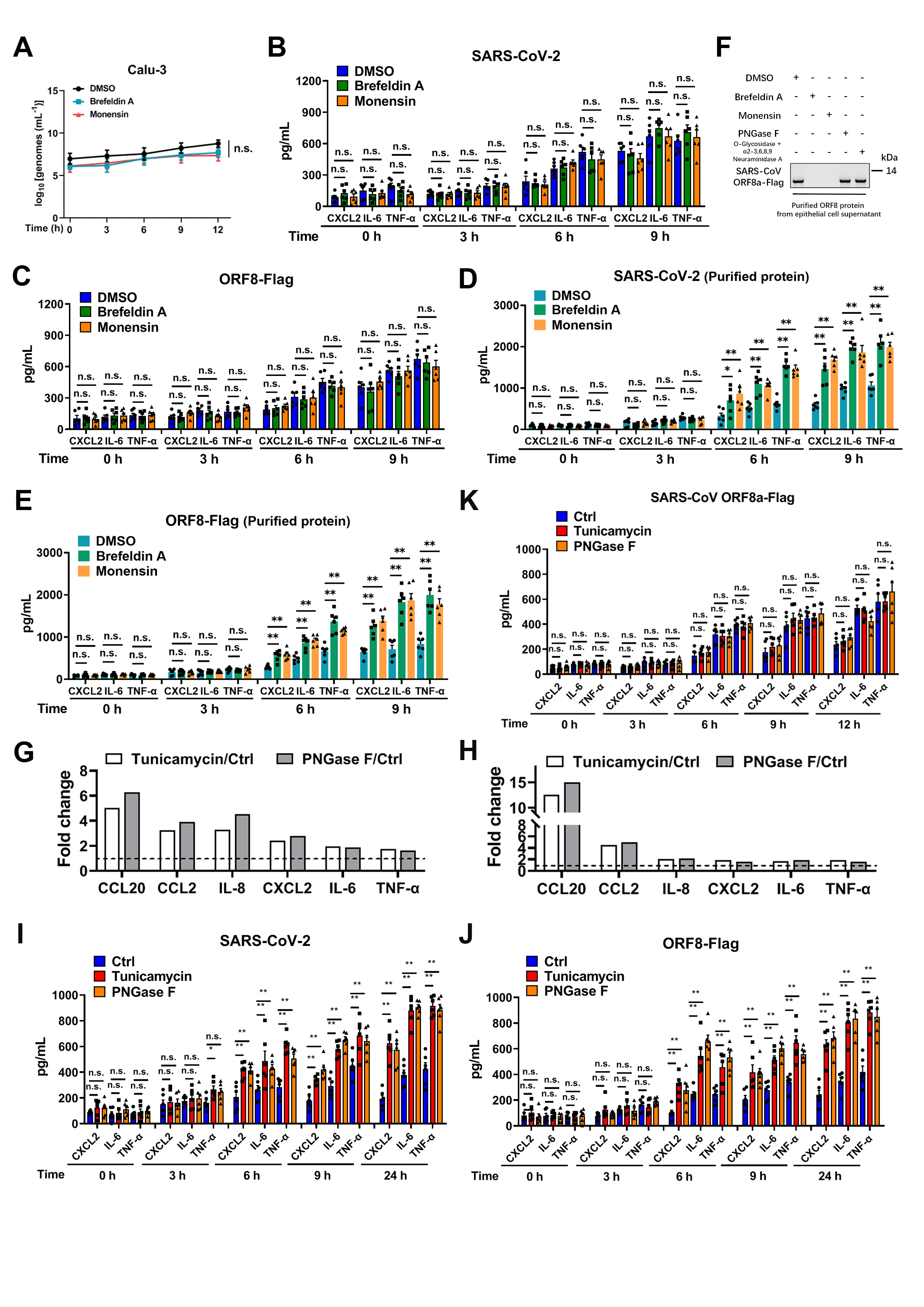

Supplement: S3 Fig — (A) Brefeldin A (3 μg/mL) or Monensin (2 μM) was used to pretreat Calu-3 cells for 2 hours, followed by SARS-CoV-2 infection at an MOI of 0.01. Cell lysates were harvested at indicated time points for detection of genomic RNA. (B-E) Brefeldin A or Monensin was used to pretreat Calu-3 cells for 2 hours, followed by SARS-CoV-2 infection (B, D), or Flag-tagged ORF8 plasmid transfection (C, E). After 12 hours, the supernatant was collected and divided into two parts. One part was used to stimulate THP-1 DM cells (B, C); the other part was used to purify ORF8 protein and then stimulate THP-1 DM cells at a final concentration of 10 ng/mL (D, E). The release of cytokines and chemokines was detected by ELISA at the indicated time points. (F) Brefeldin A or Monensinwas used to pretreat Calu-3 cells for 2 hours, followed by Flag-tagged SARS-CoV ORF8a plasmid transfection. ORF8a protein was purified and then PNGase F (1,000 units/μg protein), O-Glycosidase (4,000 units/μg protein) or α2–3, 6, 8, 9 Neuraminidase A (4 units/μg glycoprotein) was added to release glycans. Western blotting was used to detect the glycosylation of ORF8a protein. (G, H) The fold change analysis of cytokine and chemokine release in Fig 3H (G) and 3I (H). (I-K) Calu-3 cells were infected with SARS-CoV-2 (I), or transfected with ORF8-Flag (J) or SARS-CoV ORF8a-Flag plasmid (K). Tunicamycin (2μg/mL) was added into Calu-3 cells for 2 hours to prevent N-linked glycosylation; PNGase F was used to remove the N-linked glycosylation in purified ORF8 or ORF8a protein. After deglycosylation assays, purified ORF8 or ORF8a protein was used to stimulate THP-1 DM cells at a final concentration of 10 ng/mL. At the indicated time points, the release of cytokines and chemokines was detected by ELISA. Representative images from n = 3 biological replicates are shown (F). Data are shown as the mean ± s.e.m. of n = 6 biological replicates (A-E, G-K). Two-way ANOVA followed by Bonferroni post hoc test (A-E, G-K) was used for [file ppat.1011128.s003.tif]

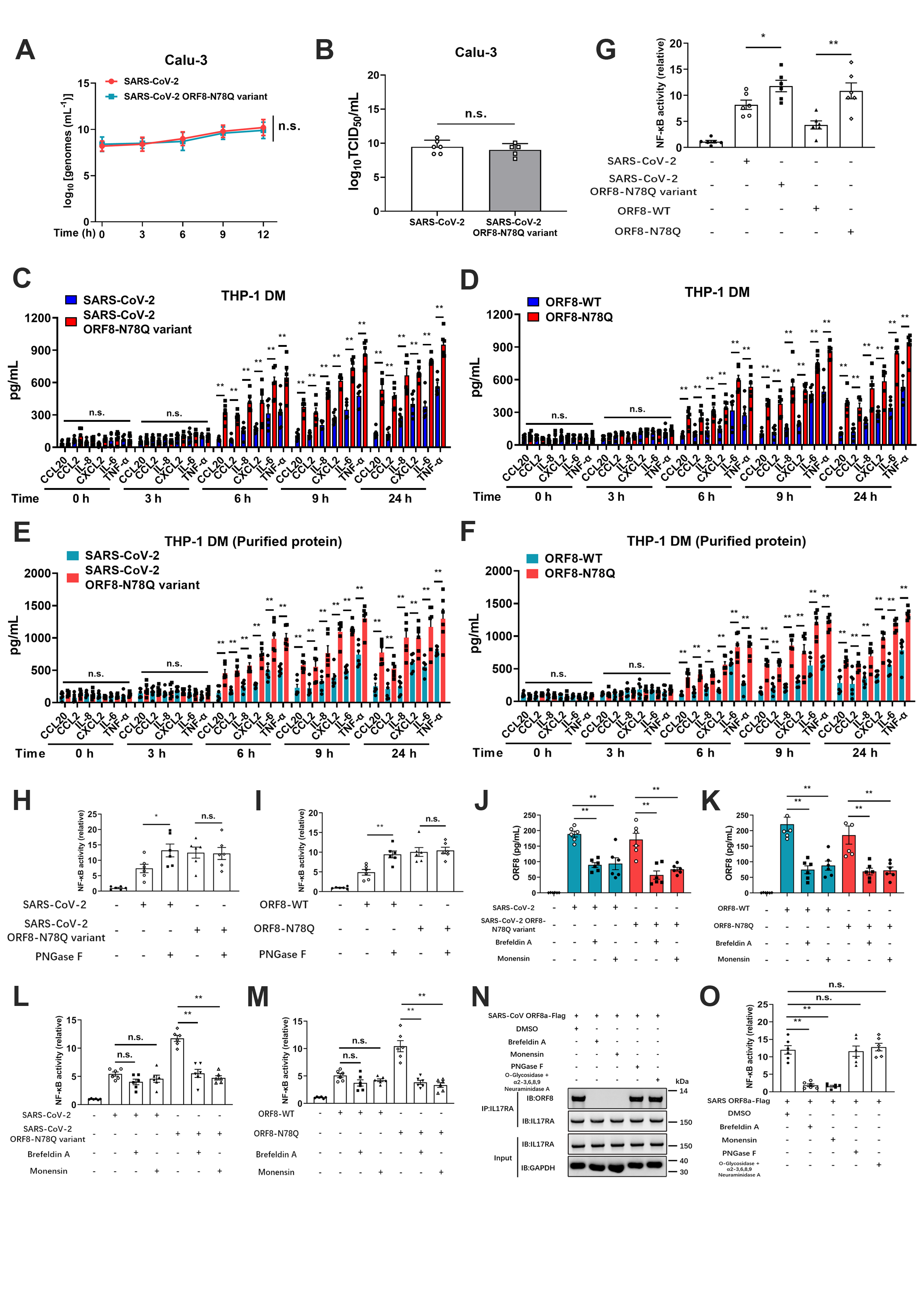

Supplement: S4 Fig — (A, B) SARS-CoV-2 or SARS-CoV-2 ORF8-N78Q variant was used to infect Calu-3 cells at an MOI of 0.01. Genomic RNA (A) and viral titers (B) were detected at indicated time points. (C-F) Calu-3 cells were infected with SARS-CoV-2 ORF8-N78Q variant (C, E), or transfected with ORF8 N78Q plasmid (D, F). After 12 hours, the supernatant was collected and divided into two parts. One part was used to stimulate THP-1 DM cells (C, D); the other part was used to purify ORF8 protein and then stimulate THP-1 DM cells at a final concentration of 10 ng/mL (E, F). The release of cytokines and chemokines was detected by ELISA at the indicated time points. (G) Calu-3 cells were infected with SARS-CoV-2 ORF8-N78Q variant, or transfected with ORF8-N78Q plasmid. The supernatant was used to stimulate THP-1 DM cells for 12 hours, the activation of IL-17 pathway was evaluated by testing NF-κB activity. (H, I) Calu-3 cells were infected with SARS-CoV-2 ORF8-N78Q variant (H), or transfected with ORF8-N78Q plasmid (I). The supernatant was collected to purify ORF8 protein. After PNGase F digestion, the ORF8 protein was used to stimulate THP-1 DM cells. After 12 hours, the activation of IL-17 pathway was evaluated by testing NF-κB activity. (J, K) Brefeldin A or Monensin was used to pretreat Calu-3 cells for 2 hours, followed by infection with SARS-CoV-2 ORF8-N78Q variant (H) or transfection with ORF8-N78Q plasmid (I). The supernatant was used to stimulate THP-1 DM cells for 12 hours. The secretion of ORF8 was detected by ELISA. (L, M) The activation of IL-17 pathway in (J, K) was evaluated by testing NF-κB activity. (N, O) Brefeldin A or Monensin was used to pretreat Calu-3 cells for 2 hours, followed by Flag-tagged SARS-CoV ORF8a plasmid transfection. ORF8a protein was purified and then PNGase F (1,000 units/μg protein), O-Glycosidase (4,000 units/μg protein) or α2–3, 6, 8, 9 Neuraminidase A (4 units/μg glycoprotein) was added to release glycans. Subsequently, ORF8a protein was used to stimulat [file ppat.1011128.s004.tif]

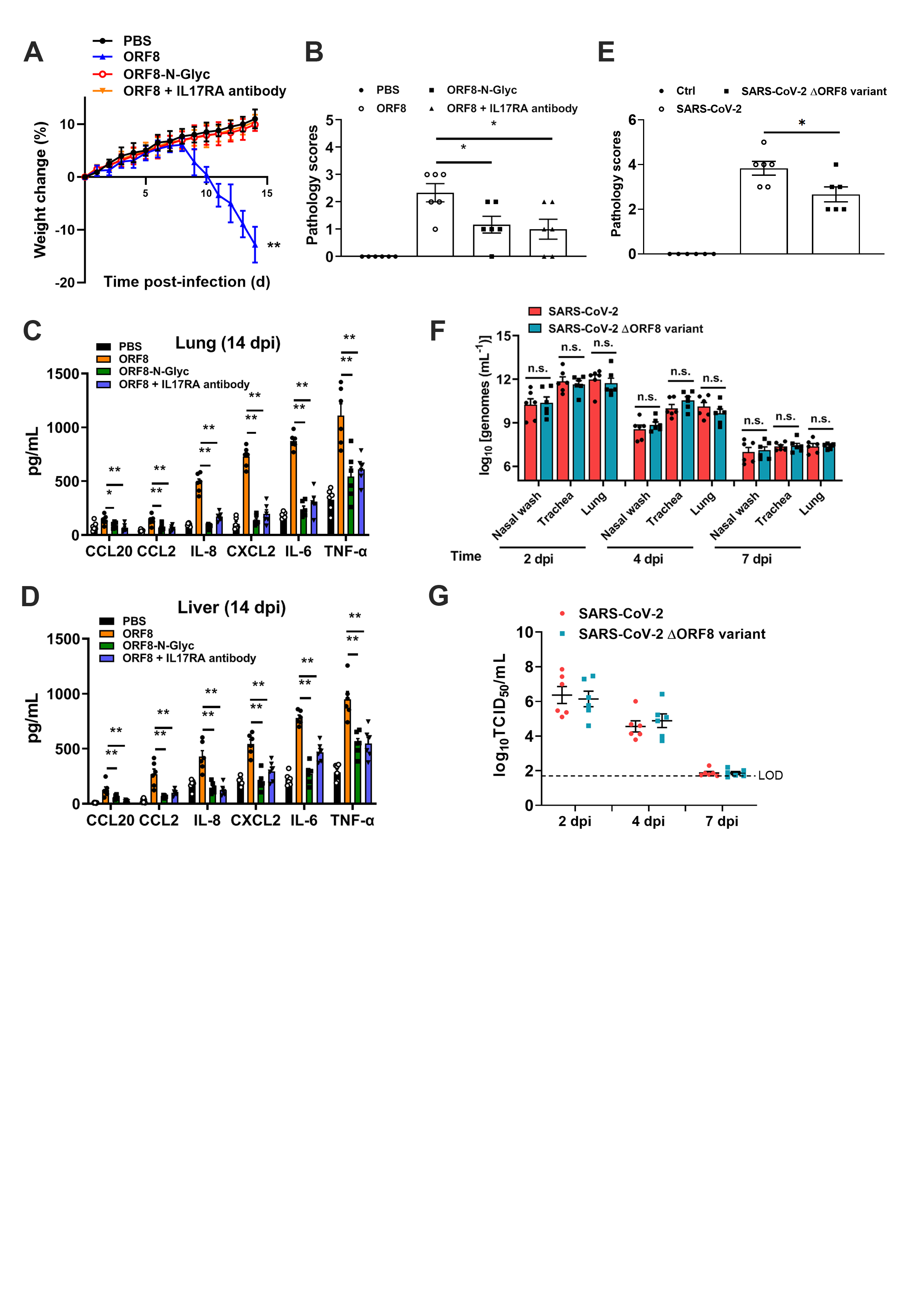

Supplement: S5 Fig — (A) Weight change of hACE2 mice treated with PBS, unglycosylated ORF8, synthetic N-linked-glycosylated ORF8 proteins, or unglycosylated ORF8 and IL17RA antibody. Data are shown as the mean ± s.e.m. (B) Histologic scoring of lungs obtained from hACE2 mice at 7 dpi in Fig 5E. (C, D) The release of cytokines and chemokines in lungs (C) and livers (D) were detected by ELISA at 14 dpi. (E) Histologic scoring of lungs obtained from hamsters at 7 dpi in Fig 5I. (F) Viral genomic RNA in nasal wash, trachea and lungs obtained from hamsters infected with SARS-CoV-2 or ORF8-deleted SARS-CoV-2 variant at 2, 4 and 7 dpi were detected by qRT-PCR. (G) Viral titers in lungs obtained from hamsters infected with SARS-CoV-2 or ORF8-deleted SARS-CoV-2 variant at 2, 4 and 7 dpi were detected by TCID50 assay. LOD, limit of detection. Data are shown as the mean ± s.e.m. of n = 6 biological replicates (B-G). One-way ANOVA followed by Bonferroni post hoc test was used for data analysis. *, p < 0.05, **, p < 0.01. Abbreviations: n.s., not significant. (TIF) [file ppat.1011128.s005.tif]
